# HSFA1a modulates plant heat stress responses and alters the 3D chromatin organization of enhancer-promoter interactions

**DOI:** 10.1038/s41467-023-36227-3

**Published:** 2023-01-28

**Authors:** Ying Huang, Jing An, Sanchari Sircar, Clara Bergis, Chloé Dias Lopes, Xiaoning He, Barbara Da Costa, Feng-Quan Tan, Jeremie Bazin, Javier Antunez-Sanchez, Maria Florencia Mammarella, Ravi-sureshbhai Devani, Rim Brik-Chaouche, Abdelhafid Bendahmane, Florian Frugier, Chongjing Xia, Christophe Rothan, Aline V. Probst, Zouine Mohamed, Catherine Bergounioux, Marianne Delarue, Yijing Zhang, Shaojian Zheng, Martin Crespi, Sotirios Fragkostefanakis, Magdy M. Mahfouz, Federico Ariel, Jose Gutierrez-Marcos, Cécile Raynaud, David Latrasse, Moussa Benhamed

**Affiliations:** 1 Université Paris-Saclay, CNRS, INRAE, Univ Evry, Institute of Plant Sciences Paris-Saclay (IPS2), 91405 Orsay, France; 2 School of Life Science, University of Warwick, Coventry, CV4 7AL UK; 3 Instituto de Agrobiotecnología del Litoral, CONICET, Universidad Nacional del Litoral, Colectora Ruta Nacional 168km 0, 3000 Santa Fe, Argentina; 4 Wheat Research Institute, School of Life Sciences and Engineering, Southwest University of Science and Technology, Mianyang, Sichuan 621010 China; 5 INRA and University of Bordeaux, UMR 1332 Biologie du Fruit et Pathologie, F-33140 Villenave d’Ornon, France; 6 GReD, Université Clermont Auvergne, CNRS, INSERM, BP 38, 63001 Clermont-Ferrand, France; 7 Laboratoire Génomique et Biotechnologie du Fruit (GBF), UMR990, INRA/INP-ENSAT, Castanet-Tolosan, France; 8 State Key Laboratory of Genetic Engineering, Collaborative Innovation Center of Genetics and Development, Department of Biochemistry, Institute of Plant Biology, School of Life Sciences, Fudan University, Shanghai, 200438 China; 9 State Key Laboratory of Plant Physiology and Biochemistry, College of Life Sciences, Zhejiang University, Hangzhou, 310058 China; 10 Department of Biosciences, Molecular Cell Biology of Plants, Goethe University Frankfurt am Main, Max-von-Laue Str. 9, 60438 Frankfurt am Main, Germany; 11 Division of Biological and Environmental Sciences and Engineering, King Abdullah University of Science and Technology, Thuwal, 23955-6900 Kingdom of Saudi Arabia; 12 Université de Paris, Institute of Plant Sciences Paris-Saclay (IPS2), F-75006 Paris, France; 13 Institut Universitaire de France (IUF), Orsay, France

**Keywords:** Chromatin structure, Plant molecular biology

## Abstract

The complex and dynamic three-dimensional organization of chromatin within the nucleus makes understanding the control of gene expression challenging, but also opens up possible ways to epigenetically modulate gene expression. Because plants are sessile, they evolved sophisticated ways to rapidly modulate gene expression in response to environmental stress, that are thought to be coordinated by changes in chromatin conformation to mediate specific cellular and physiological responses. However, to what extent and how stress induces dynamic changes in chromatin reorganization remains poorly understood. Here, we comprehensively investigated genome-wide chromatin changes associated with transcriptional reprogramming response to heat stress in tomato. Our data show that heat stress induces rapid changes in chromatin architecture, leading to the transient formation of promoter-enhancer contacts, likely driving the expression of heat-stress responsive genes. Furthermore, we demonstrate that chromatin spatial reorganization requires HSFA1a, a transcription factor (TF) essential for heat stress tolerance in tomato. In light of our findings, we propose that TFs play a key role in controlling dynamic transcriptional responses through 3D reconfiguration of promoter-enhancer contacts.

## Abstract (web summary)

Here the authors show genome-wide chromatin changes and transcriptional reprogramming occur in response to heat stress in tomato, leading to the transient formation of promoter-enhancer contacts to drive the expression of heat-stress responsive genes. Furthermore, they show chromatin spatial reorganization requires HSFA1a, a transcription factor (TF) essential for heat stress tolerance in tomato.

## Introduction

The linear conception of the genome has been replaced by an emerging understanding of the highly complex and dynamic 3D organization of chromatin within the nucleus^1–3^. Chromatin is a compact, multiscale-organized, and dynamic structure: in eukaryotes, DNA is wrapped around histones octamers (H3, H4, H2A, H2B)^4^, and this structure folds again to form chromatin loops, domains and territories, organizing the genome at multiple scales^5^. Chromosomes occupy defined territories inside the nucleus, as revealed by three-dimensional -fluorescence in situ hybridization (3D-FISH) methods and by high-throughput chromosome conformation capture (Hi-C) that show preferential interactions of each chromosome with itself^3,6^.

In mammals, chromosomes are further organized into topologically associating domains (TADs), genomic regions that preferentially form contacts with each other rather than with surrounding sequences. TADs have been proposed to allow coordinated regulation of genes situated inside the TAD by allowing contact with distal regulatory sequences with in the TAD and limiting their access to sequences outside of the TAD; although this model is still under debate^7^. TAD associations form “chromatin compartments”. Active (A) compartments represent active chromatin defined by active histone marks, a high density of genes, and an elevated transcription rate supported by high RNA polymerase II (RNAPII) activity. In contrast, B compartments represent inactive chromatin defined by repressive histone marks, DNA methylation, and an increased number of transposable elements^8,9^. At a smaller scale, the formation of chromatin loops brings together distal regulatory sequences with promoter regions, or isolates them from promoters^10^. These short and long-range interactions modulate the expression of neighbouring and distant genes^11^. Chromatin loop may involve self-interacting transcription factors (TFs), that bring together the genome regions they bind^12,13^.

All these organizational levels have been defined in animals and observed in plants: A and B compartments alternate along the genome, with A compartments predominantly in telomeric regions and B compartments in pericentromeric regions in various crop species^9,14^. However, plant chromatin organization shows several differences from that in animals. First, plant TAD-like structures represent only about 25% of the rice (*Oryza sativa*) genome^15^ compared to 75% for humans^16^. However, this low number of TADs in rice be a consequence of how we currently define TADs and may not reflect the characteristics of plant genomes^17^. Likewise in wheat (*Triticum aestivum*), TAD-like structures correspond to large heterochromatin domains highly enriched in transposable elements, rather than to groups of co-regulated genes^3^. The term “intergenic condensed spacers” (ICONS) has been proposed to describe these structures, as they seem to condense silent chromatin to allow loop formation between genes located outside of the ICONS, forming transcription factories. Second, mechanisms controlling chromatin folding differ between plants and animals. In animals, cohesin complexes extrude chromatin via their ring-like shape until the chromatin binds at two convergent CTCF proteins at specific CTCF binding sites that define TAD boundaries^18^. However, plant genomes apparently lack CTCF homologues, therefore how TAD-like domains are structured in plants is unclear.

Although the overall structure of chromatin architecture is very robust across organs and growth conditions, detailed analyses of chromatin folding revealed it can be modulated during development^19^, depending on cell type^20^ or in response to changes in environmental conditions^21^. In plants, some changes in chromatin architecture in response to various stresses likely coordinate global transcriptome modifications for appropriate cellular and physiological responses. The importance of chromatin conformation for gene regulation in response to environmental stress in *Arabidopsis thaliana* has been well documented^21,22^. However, whether and how stress induces chromatin reorganization in crops is less understood, and only a few studies have examined the plant response to temperature changes. Rice chromosomes decondense under cold stress, decreasing long-range interactions above 1 Mb and increasing A-B compartment interactions^15^. Conversely, exposure to heat stress reduces short-range interactions between A-B compartments, as A-A and B-B compartment interactions decrease^15^.

Gene expression is also controlled by establishment or dissociation of interactions between distal regulatory elements (REs) and promoters, such as enhancer-promoter interactions. In plants, such long-range interactions between promoters and distal regulatory sequences are most prominent in species with large genomes, such as maize (*Zea mays*)^23,24^. Modulating these interactions in response to stress is likely functionally relevant, as has been shown in Drosophila^25^ and human cells^26^, but remains largely unexplored in plants.

Here, we studied the chromatin-based regulation of RE-promoter contacts and how nuclear DNA reorganization affects gene expression. To this end, we captured the temporal dynamics of these contacts using a time-course analysis in tomato (*Solanum lycopersicum*) during heat stress (HS). We find that HS induces profound changes in chromatin accessibility, as well as dynamic interactions between promoters and distal REs. We further show that the Heat Shock Factor (HSF) TF HSFA1a, a master transcriptional regulator of heat stress responses in plants^27^, plays a key role in the dynamic formation of promoter-enhancer contacts in response to heat at several loci. Together, these results allow us to propose a model in which changes in chromatin accessibility and binding of HSFA1 promote the formation of promoter-enhancer contacts to induce gene expression in response to HS.

## Results

### Tomato chromatin is highly compartmentalized and organized around transcription factories

**Fig. 1:**
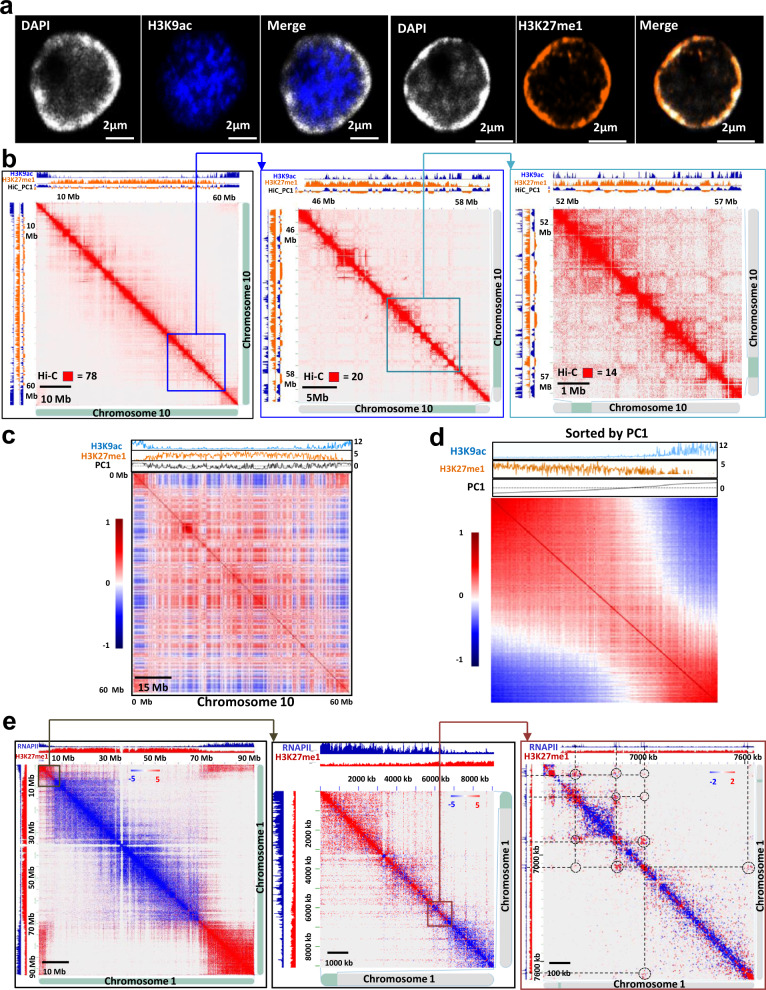
Tomato chromatin architecture displays a strong compartmentalization and is organized around transcription factories. **a** Immunofluorescence detection of H3K9ac (blue) and H3K27me1 (orange) histone modifications and DAPI staining (grey) in an isolated tomato nucleus. 3 times each experiment was repeated independently with similar results. **b** Chromosome 10 Hi-C interaction matrix at increasing levels of resolution. H3K9ac ChIP-seq signal (blue) and H3K27me1 signal (orange) were aligned with the maps to highlight the correlation with compartmentalization in tomato. **c** Pearson correlations of distance-normalized Hi-C interaction frequency maps of chromosome 10. The ChIP-seq signal for H3K9ac (blue) and H3K27me1 (orange) as well as the -PC1 component from principal component analysis (black) were aligned to the map. **d** Previous interaction matrix with reorganized bins according to their PC1 value. **e** Chromosome 1 interaction matrix obtained by calculating the difference between the RNAPII HiChIP and the Hi-C signal. Red dots are interactions enriched in RNAPII HiChIP compared to Hi-C, and blue dots are interactions enriched in Hi-C compared to RNAPII HiChIP.

To explore tomato chromatin architecture, we performed an immuno-detection experiment on interphase somatic nuclei using antibodies directed against two different histone modifications, histone H3 lysine 9 acetylation (H3K9ac) and histone H3 lysine 27 monomethylation (H3K27me1), associated with transcriptionally active chromatin and constitutive heterochromatin, respectively. Immuno-staining revealed these marks were not homogeneously distributed in the nucleus, indicating distinct euchromatin and heterochromatin compartments in tomato: euchromatin occurred in the centre of the nucleus while constitutive heterochromatin occurred at the nuclear periphery (Fig. 1a, Supplementary Fig. 1).

To provide a high resolution view of chromatin architecture in tomato, we performed Hi-C, a genome-wide method to detect DNA-DNA physical interactions^8^. We observed a strong signal on the main diagonal, indicating frequent interactions between adjacent loci (Fig. 1b). The Hi-C map also revealed a strong compartmentalization segregating euchromatin and constitutive heterochromatin. To assess the ability of a genomic region to form a specific type of compartmental domain with specific epigenetic features, we generated a Pearson correlation map sorted it by multiple chromatin landscapes. To that end, we reordered the rows and columns of the correlation matrix: instead of arranging according to their position along the linear sequence, we arranged bins by increasing signal of the chosen feature. Notably, when sorted according to PC1 values (discriminating between A and B compartments), the matrix displayed clear segregation of chromosome 10 into two types of compartmental domains (Fig. 1c, d). These results highlight that histone marks strongly correlate with and are predictive of compartmental organization in tomato. In addition, integrating the Hi-C interaction matrix with RNAPII chromatin immunoprecipitation combined with sequencing (ChIP-seq) at different resolutions revealed interaction hotspots between genomic bins containing actively transcribed genes, indicating that tomato chromatin is organized around transcription factories (Supplementary Fig. 2). We performed immunostaining experiments using an anti-RNAPII antibody and observed RNAPII foci (Supplementary Fig. 1b). To further validate the presence of transcription factories, we performed a HiChIP experiment, a sensitive method to analyze protein-centric chromosome conformation, using an anti-RNAPII antibody^3^. These analyses confirmed that active chromatin in tomato is largely organized around transcription factories (Fig. 1e), as previously described in wheat^3^.

### Heat stress strongly affects global chromatin organization

To understand the impact that environmental stress has on tomato chromatin 3D organization and gene expression, we focused our analysis on HS, which affects both components in different plant species^22,28^. To this end, we exposed tomato plants to high heat for 1 and 6 hours (Supplementary Fig. 3), these time points were selected based on the expression of the HS early response gene *HSFA2* that peaked after 1 h of exposure and returned to basal level after 4 to 8 h (Supplementary Fig. 4). We next performed RNA-seq to obtain a global view of the changes in gene expression induced by HS. We found that compared to control conditions, one hour of HS induced the expression of genes known to be involved in HS responses (Supplementary Fig. 5a). To visualize dynamic gene expression patterns, we applied an unsupervised, self-organizing map (SOM) machine-learning approach^29^ to reveal correlations between differentially expressed genes (DEGs) in multiple treatments (Supplementary Fig. 5b). Notably, this analysis revealed that a large part of DEGs after one hour of HS reverted their expression to their original expression levels after six hours (Supplementary Fig. 5c, d). To determine the extent to which chromatin architecture is affected by HS in tomato, we measured nuclei size after 0, 1, 6 h of HS and found that nuclei increased size after heat treatment (Supplementary Fig. 6).

**Fig. 2:**
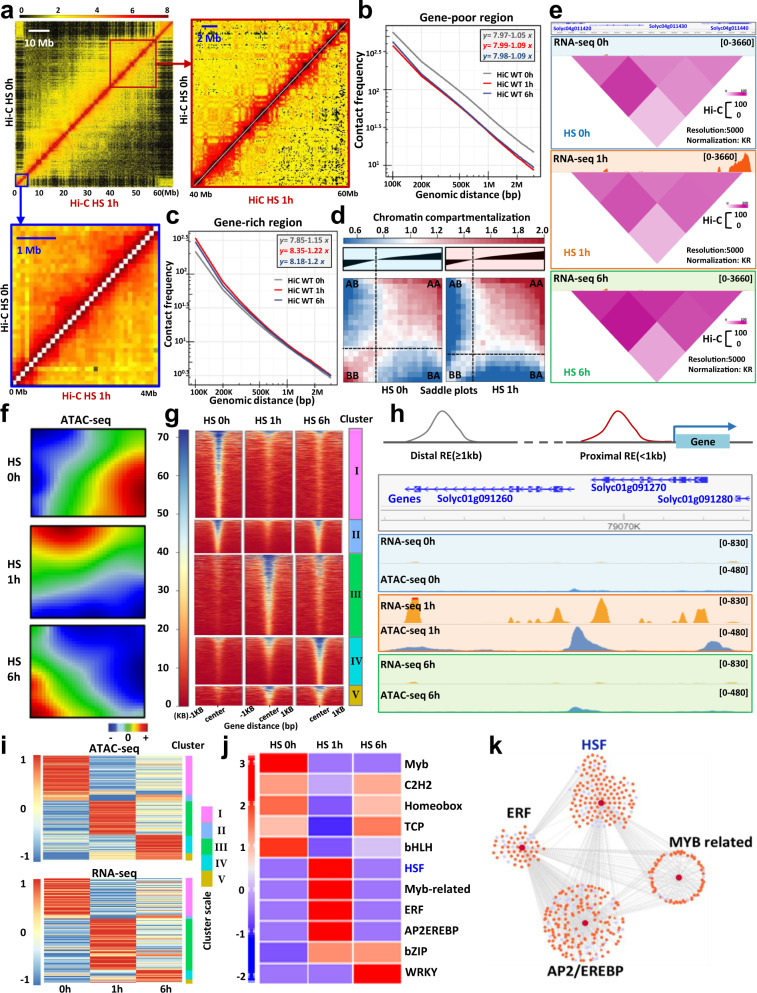
The high-order tomato chromatin organization is strongly affected by heat stress. **a** Chromosome 10 Hi-C interaction frequency heatmap at a 100-kb resolution in control and heat stress conditions (1 h). Each pixel denotes all interactions between any to 100 kb genomic loci from the linear genome. Intensity represents log_2_ normalized contact frequencies. **b** Averaged scaling plot of interaction frequencies against increasing genomic distance for all tomato pericentromeric regions (gene poor region). The genomic bin size is 100 kb. **c** Averaged scaling plot of interaction frequencies against increasing genomic distance for all tomato chromosome arms (gene-rich regions). The genomic bin size is 100 kb. **d** Saddle plots of Hi-C data representing the mean observed interaction frequencies divided by expected interaction frequencies between 20 kb bins. Interactions between A compartments are in the top right, and interactions between B compartments are in the bottom left. **e** Hi-C interaction heat maps at a 5 kb resolution surrounding a genomic region with differential gene expression along the time course analysis. **f** Self-organizing map (SOM) of chromatin accessibility profiles at 0, 1 and 6 h of heat stress. Red areas mark over-accessible chromatin sites, blue areas mark under-accessible chromatin sites. **g** K-means clustering of differentially accessible peaks. **h** Example of proximal regulatory elements (REs) showing correlation between RNA-seq signals and ATAC-seq signals along the time course. **i** Correlation between ATAC-seq and RNA-seq data shown by heatmaps of proximal accessible chromatin sites and corresponding gene expression levels. **j** HOMER DNA-motif enrichment analyses of proximal accessible chromatin peaks. **k** TF network built by integration of RNA-seq and ATAC-seq data. Source data of Fig. 2g are provided as a Source Data file.

To test if chromatin architecture reorganization and gene expression changes under heat stress are correlated, we performed Hi-C experiments using the same conditions employed for the transcriptomic analysis. To define the effects of HS on chromatin architecture, we measured the relative interaction differences at a 100-kb resolution (Fig. 2a). Visual inspection revealed that in plants exposed to HS, the interactions within constitutive heterochromatin were reduced whereas almost no changes were observed within euchromatin. To quantify these effects, we generated scaling plots of interaction frequencies against genomic distance at a 100-kb resolution on control and heat stressed plants. We found that HS caused a decrease of chromatin interactions in constitutive heterochromatin regions whereas chromatin interactions in euchromatin were enhanced (Fig. 2b, c, Supplementary Fig. 7). These observations suggest that heat negatively affects the strength of the compartmentation in constitutive heterochromatin, and positively affects it in euchromatin. Nevertheless, we cannot rule out the possibility that the slight increase in the number of interactions detected in euchromatin could be a mere consequence of the reduction in heterochromatin interactions, which would mechanically increase the sequencing depth for euchromatic interactions. To test this, we generated saddle plots to measure the degree to which A (euchromatin) and B (constitutive heterochromatin) compartments segregate in the nucleus. We found that HS positively affected the A–A interactions and negatively affected the B–B interactions (Fig. 2d, Supplementary Fig. 8). However, we integrated RNA-seq and Hi-C data at a 5 kb resolution across the HS time course and found that HS DEGs resided in domains in which the resolution of our Hi-C does not allow to observe major changes (Fig. 2e), suggesting that regulatory dynamics may occur via intradomain contacts. To test this hypothesis, we used ATAC-seq which identifies accessible chromatin sites to identify putative REs that may physically interact with promoters of HS DEGs (Fig. 2f–k, Supplementary Fig. 9). To visualize the dynamics of chromatin accessibility patterns across the entire HS time course, we applied an unsupervised, SOM machine-learning approach^29^ (Fig. 2f). The chromatin accessibility pattern was dynamically modified by heat stress and exhibited temporal changes in RE activation. Since these regulatory sequences can be hundreds to thousands of base pairs upstream or downstream of the gene they regulate, a simple association between chromatin accessibility and gene expression may not be always accurate. Therefore, we thus classified all the accessible chromatin peaks into two categories: those within 1.0 kb upstream of transcription start sites (TSS-proximal) and those found further away from genes (TSS-distal). We focused our analysis on TSS-proximal peaks. Using a clustering approach based on the level of chromatin accessibility, we identified five clusters (I to V) (Fig. 2g). Chromatin accessibility is necessary, but not sufficient, to induce gene expression, which is also determined by histone modifications and the availability of recruited TFs^30^. We found that the accessibility of proximal REs correlated positively with gene expression (Fig. 2h, i). The ATAC-seq time-course analysis can uncover sequential TF binding based on characteristic chromatin footprints. We therefore used the accessible chromatin data from the HS time course to determine the hierarchical gene regulation networks modulated by heat (Fig. 2i). We found that Heat Stress Transcription Factors (HSF) and other TF families (ERF, Myb-related and AP2) were upregulated only shortly after HS (Fig. 2j) while that TFs from the C2H2, Homeobox and TCP families were downregulated after 1 hour of HS. Moreover, we generated hierarchical TF networks by taking into account changes in gene expression as well as the presence of specific TF binding sites in their promoter region. We found that the chromatin accessibility at specific TF-binding motifs, which reflects the TFs activity, coincided with altered expression of genes harbouring an accessible binding site for this TF in their promoter (Fig. 2k). Altogether, our analyses suggest that sequential activation of TFs controls gene expression during the heat stress response via increased chromatin accessibility: a first wave of transcriptional changes could be controlled by HSF and ERF factors, and would involve early response event, and a second wave would subsequently occur under the control of WRKYs and TCPs.

### Distal and proximal REs display different chromatin signatures

**Fig. 3:**
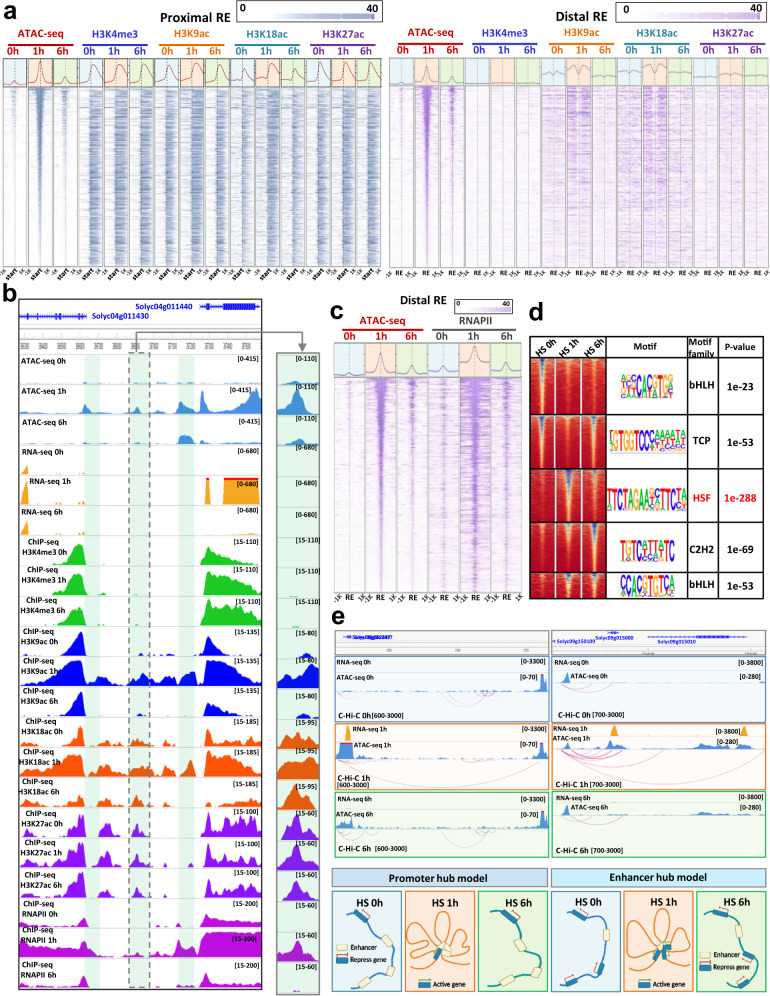
Distal and proximal REs displayed different chromatin signatures. **a** Heatmaps of ATAC-seq and ChIP-seq (H3K4me3, H3K9ac, H3K18ac and H3K27ac) profiles over proximal (grey) and distal (purple) regions at 0, 1 and 6 h of heat stress. **b** Example of a distal regulatory element (RE) and of the histone modifications surrounding it after 0, 1 and 6 h of heat stress. **c** Heatmaps of ATAC-seq signal and RNAPII occupancy over distal REs at 0, 1 and 6 h after heat stress. **d** K-means clustering of distal differentially accessible chromatin regions and the corresponding top enriched motif identified by HOMER (v4.11) for each cluster. Binomial distribution test was used in detected ATACseq peaks. **e** Example of two types of transient chromatin loop formation: a promoter-centric hub and a RE-centric hub. Significant interactions for C-Hi-C and HiChIP-RNPII data were detected with HOMER (v4.11) using cumulative binomial distribution with p value 0.05.

Active REs are associated with specific histone modifications in mammals (i.e. H3K4me1 and H3K27ac)^31,32^. To determine if a typical chromatin signature differentiates distal and proximal REs in tomato, we performed ChIP-seq under control and HS conditions and analyzed the levels of four histone covalent modifications that have been previously associated with active transcription: H3K4me3, H3K9ac, H3K18ac and H3K27ac. We found that the accumulation of histones with these modifications did not vary significantly under HS (Supplementary Fig. 10). However, we found that nucleosomes flanking accessible proximal REs displayed a high level of H3K9ac, H3K18ac, H3K27ac and H3K4me3 (Fig. 3a, b, Supplementary Figs. 11–13). In contrast, nucleosomes flanking accessible distal REs displayed a high level of H3K9ac and H3K18ac, a moderate level of H3K27ac and low level H3K4me3 (Fig. 3a, b). These data, in combination with chromatin accessibility data, suggested that nucleosomes decorated with H3K9ac and H3K18ac and devoid of H3K4me3 may indicate active enhancer positions in the genome, and could be used to annotate enhancer regions.

In mammals, it has been reported that distal enhancers recruit RNAPII^33^. We postulated that this feature could also be present at distal active enhancers in plants. To test this hypothesis, we performed RNAPII ChIP-seq on tomato plants subjected to HS and analyzed its accumulation on distal REs specifically active at 1 hour after HS. We identified a large number of RNAPII binding sites near TSSs, but also present at the distal REs identified by our chromatin analyses (Fig. 3c, Supplementary Figs. 14, 15). We then determined motifs associated with these HS-activated distal REs and found that both proximal and distal elements contained motifs for the same TFs, suggesting that the same TFs could bind both proximal and distal REs (Fig. 3d).

### 3D chromatin reorganization transiently induces promoter-RE interactions in response to HS

To determine whether distal REs interact with promoters, we further combined Hi-C with a capture enrichment step (C-Hi-C), a powerful tool to delineate spatial and functional relationships at specific chromatin regions. To this end, we generated a biotinylated RNA bait library specifically targeting 212,735 promoter regions (Supplementary Data 1) and performed a C-Hi-C across the HS time course to reveal the different chromatin associations during HS. As expected, C-Hi-C enables the identification of a large number of chromatin contacts, increasing the resolution of our analysis (Supplementary Fig. 16). Interestingly, we found that in addition to the classical 1 vs. 1 RE-promoter contacts, we observed two types of interactions implicating multiple loci: (i) one promoter interacted with several REs, called “promoter-centric hubs”, and (ii) one RE interacted with several genes, named “RE-centric hubs” (Fig. 3e). To further dissect the properties of RE-promoter contacts, we carried out a differential analysis integrating interaction data from control and HS treatments. This analysis revealed notable dynamic changes between the different types of RE-promoter contacts (Fig. 3e, Supplementary Fig. 17, Supplementary Data 2). Distal REs are thought to be brought into physical proximity to their target promoters via the three-dimensional looping of chromatin mediated by structural proteins such as TFs^34,35^. Therefore, we hypothesized that the strength of a RE-promoter chromatin interaction could be linked to the accessibility of both the RE and the promoter to TFs. To test this, we classified the promoter-associated interactions into three categories: (i) interactions for which neither anchor is not accessible, (ii) interactions for which only one anchor is not accessible, and (iii) interactions for which both anchors are accessible. The strength of interactions in which both anchors are accessible was stronger than the interactions in which only one anchor or no anchors are accessible (Supplementary Fig. 18). Thus, HS induces transient promoter/RE interactions that correlate with an increase in chromatin accessibility at both sites. Together with the observation that both proximal and distal REs are enriched in the same TF binding motifs points these results point to a putative role of DNA binding proteins such as TFs to promote or stabilize these interactions.

**Fig. 4:**
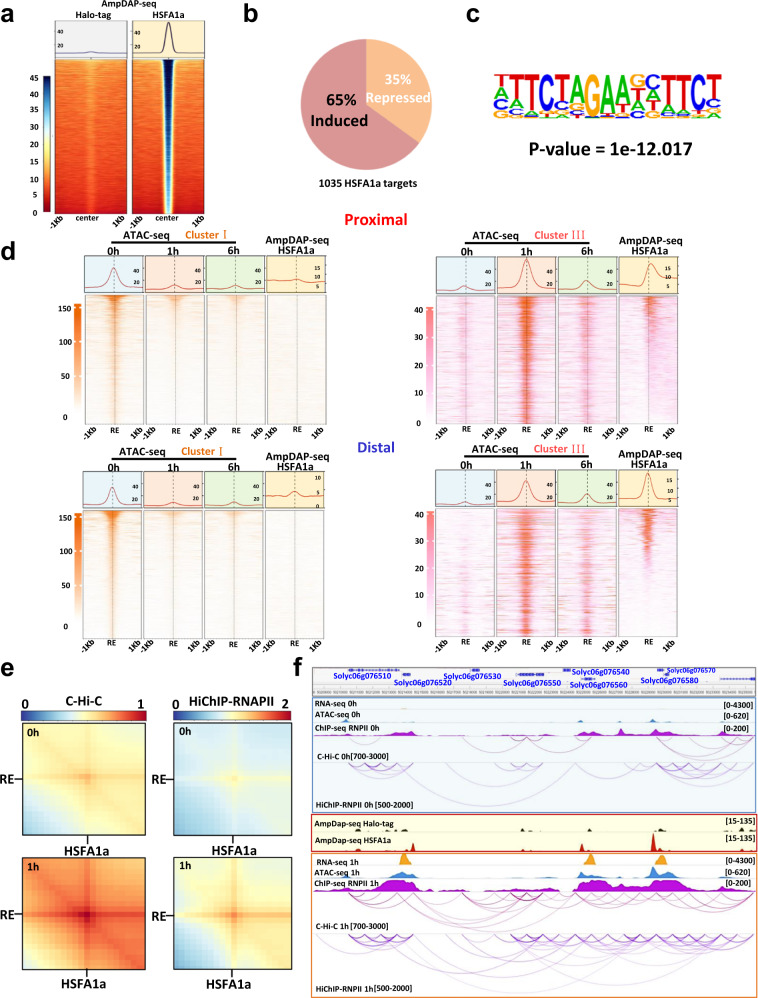
HSFA1a binding sites correlate with anchors of transient promoter-enhancer chromatin loops formed in response to heat stress. **a** Heatmaps of control Halo-tag and HSFA1a density in the region of ±1 kb around the HSFA1a peaks. **b** Proportion of HSFA1a targets that were mis-regulated after 1 h of heat stress compared to the control condition; 65% of HSFA1a targets were upregulated after 1 h of heat stress. **c** Top enriched motif in HSFA1a peaks identified by HOMER (v4.11). Binomial distribution test was used in detected DAPseq peaks. **d** Heatmaps of DAP-seq and ATAC-seq profiles surrounding proximal (top) or distal (bottom) regions accessible only in the control condition (left) or accessible only after 1 h of heat stress (right). **e** Aggregate plots quantifying the mean of aggregated/stacked C-Hi-C submatrices (left) or RNAPII HiCHIP (right) between HSFA1a and distal regulatory element (RE) loops after an observed/expected transformation of the matrix, for control and heat stress conditions. **f** A connection between gained contacts and induction of heat-related genes is exemplified. DAP-seq Halo-tag signal is represented in black and HSFA1a in red. RNA-seq signals are represented by orange peaks, ATAC-seq signals by blue and ChIP-seq signal of RNAPII by purple peaks. Chromatin interactions identified by C-Hi-C are represented by red lines and HiChIP RNAPII by purple lines. Significant interactions for C-Hi-C and HiChIP-RNPII data were detected with HOMER (v4.11) using cumulative binomial distribution with *p*-value 0.05.

Our integrative analyses revealed that the HSF binding motif was over-represented among the REs involved in the promoter-enhancer interactions only after 1 h of HS treatment. The first exhaustive overview of HSF family in plants was described in *Arabidopsis*, where 21 representatives were identified, belonging to three classes (A, B, and C) according to the structural features of their oligomerization domains^36^. Class A HSFs are essential for transcriptional activation, whereas class B and C HSFs have no activator function because they lack the acidic amino acid residue containing motifs^37^. Previous studies have shown that HSFA1a is the master regulator of the transcriptional HS primary response^27,38,39^. Interestingly, previous work in *S. lycopersicum* showed that HSFA1a is both a master regulator of the HS response and basal thermo-tolerance^38^. We thus hypothesized that HSFA1a may be implicated in RE-Promoter interactions during early HS responses. To test this, we first determined HSFA1a binding sites using a TF-DNA binding assay called DNA affinity purification and sequencing (DAP-seq)^40^. This approach revealed a large number (5034) of putative HSFA1a binding sites (Fig. 4a, Supplementary Fig. 19). As expected, these binding sites were preferentially found in the immediate vicinity of the TSS of annotated genes (Supplementary Fig. 20) which were associated with 1,035 HS DEGs (Fig. 4b, Supplementary Fig. 21). Interestingly, a significant part of these potential binding sites was found in chromatin regions that became transiently accessible 1 h after HS (Supplementary Fig. 22). Implementing a de novo motif discovery analysis we found that the HSFA consensus sequence (TCTAGAANNTTCT) was over-represented within the HSFA1a DAP-seq peaks (Fig. 4c). Integrating DAP-seq and transcriptomic data revealed that 65% of the putative HSFA1a target genes that were differentially expressed after 1 h of HS were up-regulated whereas 35% were down-regulated supporting the view that HSFA1a acts primarily as a transcriptional activator (Fig. 4b). Next, we explored the relationship between chromatin accessibility and HSFA1a-enriched loci by integrating ATAC-seq and DAP-seq data (Fig. 4d). A significant proportion of chromatin regions accessible after 1 h of HS displayed HSFA1 binding, whereas this was not the case in chromatin regions that were accessible in control conditions. We then tested whether the strength of spatial contacts between HSFA1a binding sites and distal REs differed in heat stress vs. control conditions. To test this idea, we generated aggregate plots (aggregated C-Hi-C matrices) to quantify the mean of aggregated/stacked C-Hi-C submatrices between HSFA1a–distal RE loops after an observed/expected transformation of the C-Hi-C matrix, for control and HS conditions. These analyses revealed an increase of the strength of the HSFA1a-distal RE contacts after 1 h of HS compared to the control (Fig. 4e, left). Since we previously observed that many distal REs were bound by RNAPII, we performed a HiChIP experiment with an anti-RNAPII antibody to detect and measure the strength of the HSFA1a-distal RE interactions. This analysis revealed an increase of the number and strength of the distal RE-HSFA1a contacts after 1 h of HS compared to the control (Fig. 4e, f). Taken together, our data suggest that HSFA1a plays a major role in RE-promoter interactions.

### HSFA1a plays a major role in the dynamic formation of promoter-enhancer contacts in response to HS

**Fig. 5:**
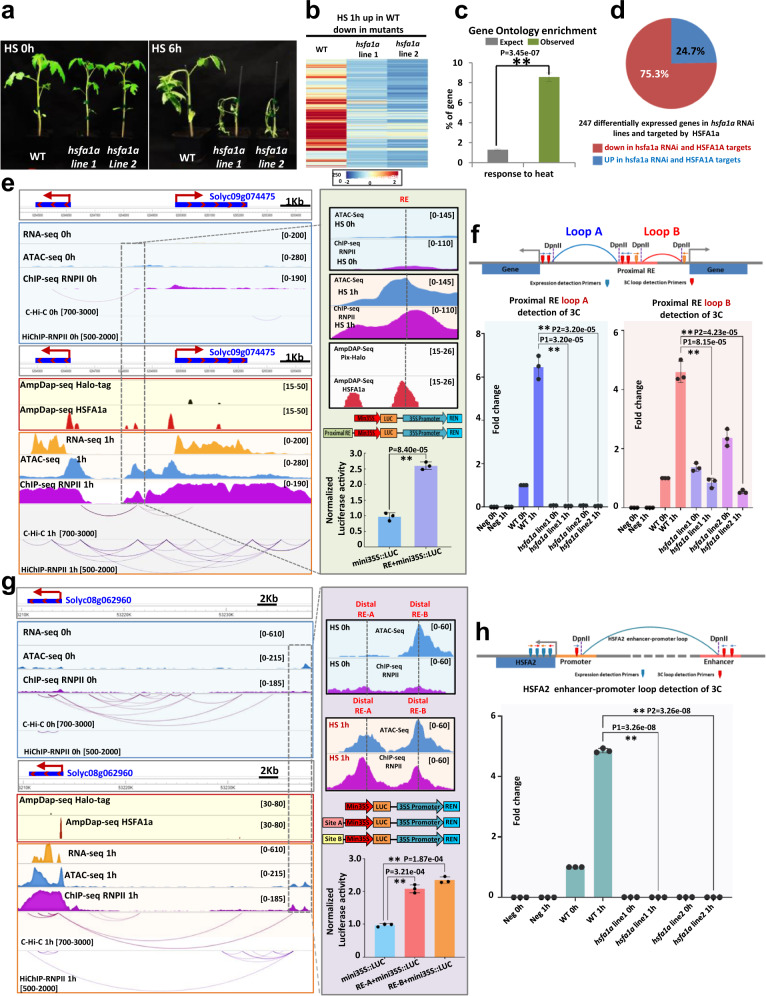
HSFA1a plays a major role in the dynamic formation of promoter-enhancer contacts in response to a heat stress. **a** Phenotypes of WT and *hsfa1a* knock-down (via RNA interference) lines after 0 and 6 h of heat stress. **b** Expression level heatmap of genes that were down-regulated in two independent *hsfa1a* lines after 1 h of heat stress compared to the WT. **c** Gene ontology enrichment of down-regulated genes (*n* = 280). P-values calculated using Fisher’s exact test. **d** Pie chart representing the 247 HSFA1a target genes that were mis-regulated in *hsfa1a* lines after 1 h of heat stress compared to the WT. Among them, 75.3% were down-regulated in *hsfa1a* compared to the WT. **e**, **g** Screenshot of the *Solyc09g074475* and *HSFA2* locus. DAP-seq against Halo-tag and HSFA1 are represented in black and red. The RNA-seq, ATAC-seq and RNAPII ChIP-seq signal is represented in orange, blue and purple respectively. Chromatin interactions from C-Hi-C data and RNAPII HiChIP are represented by red and purple lines. The right green box of **e** shows a zoom of a distal RE that interacts with two heat stress-responsive genes (*Solyc09g074475* and one non-annotated gene). The purple box of **g** shows a zoom of two distal REs named A and B that interact with *HSFA2*. The histogram represents the luciferase activity from a transient luciferase assay using a *RE-mini35S::LUC* construct or the *mini35S::LUC* alone as a control. The mean LUC/REN activity levels were normalized to *mini35S::LUC* as control (*n* = 3 biological replicates). **f**, **h** (Top) Design of the 3C-qPCR assays used to analyze the distal RE loops in the WT and *hsfa1a* lines in response to heat stress. (Bottom) Quantitative 3C; the relative interaction frequencies were calculated as described in the Materials and Methods. Data are the average of three biological replicates each with three technical replicates (*n* = 3). Circles in 5e, g denote relative LUC/REN activity values. Circles in **e**, **f**, **g**, **h** denote relative interaction frequency. Bars indicate mean values + /− SD of three replicates. Exact *P*-values are shown (two-tailed, two-sample Student’s *t*-test). Source data are provided as a Source Data file.

To determine if the dynamic formation of chromatin loops induced by HS is dependent on HSFA1a activity, we undertook a genetic approach. We used two independent *hsfa1a* knock-down transgenic lines (*hsfa1a-line1 and hsfa1a-line2*) carrying a hairpin RNA construct that downregulates *HSFA1a* expression^27^ (Supplementary Fig. 23). In accordance with previous studies, we found that compared to wild type (WT) plants, *hsfa1a* knock-down transgenic plants displayed strong defects in growth when exposed to HS (Fig. 5a). To determine the role of HSFA1a on HS-mediated gene expression, we performed a transcriptomic analysis on control and *hsfa1a* knock-down transgenic lines subjected to 1 h of HS (Fig. 5b). Gene ontology analysis on down-regulated genes in *hsfa1a* knock-down transgenic lines compared to WT plants revealed significant enrichment in genes involved in the HS response (Fig. 5c). We found that 247 of the HSFA1a targets identified by DAP-seq were misregulated in *hsfa1a* knock-down transgenic lines, 186 (75.3%) being downregulated and 61 (24.7%) upregulated (Fig. 5d and Supplementary Fig. 24). This data reinforces the view that HSFA1a acts as a positively regulator of gene expression in response to HS.

**Fig. 6:**
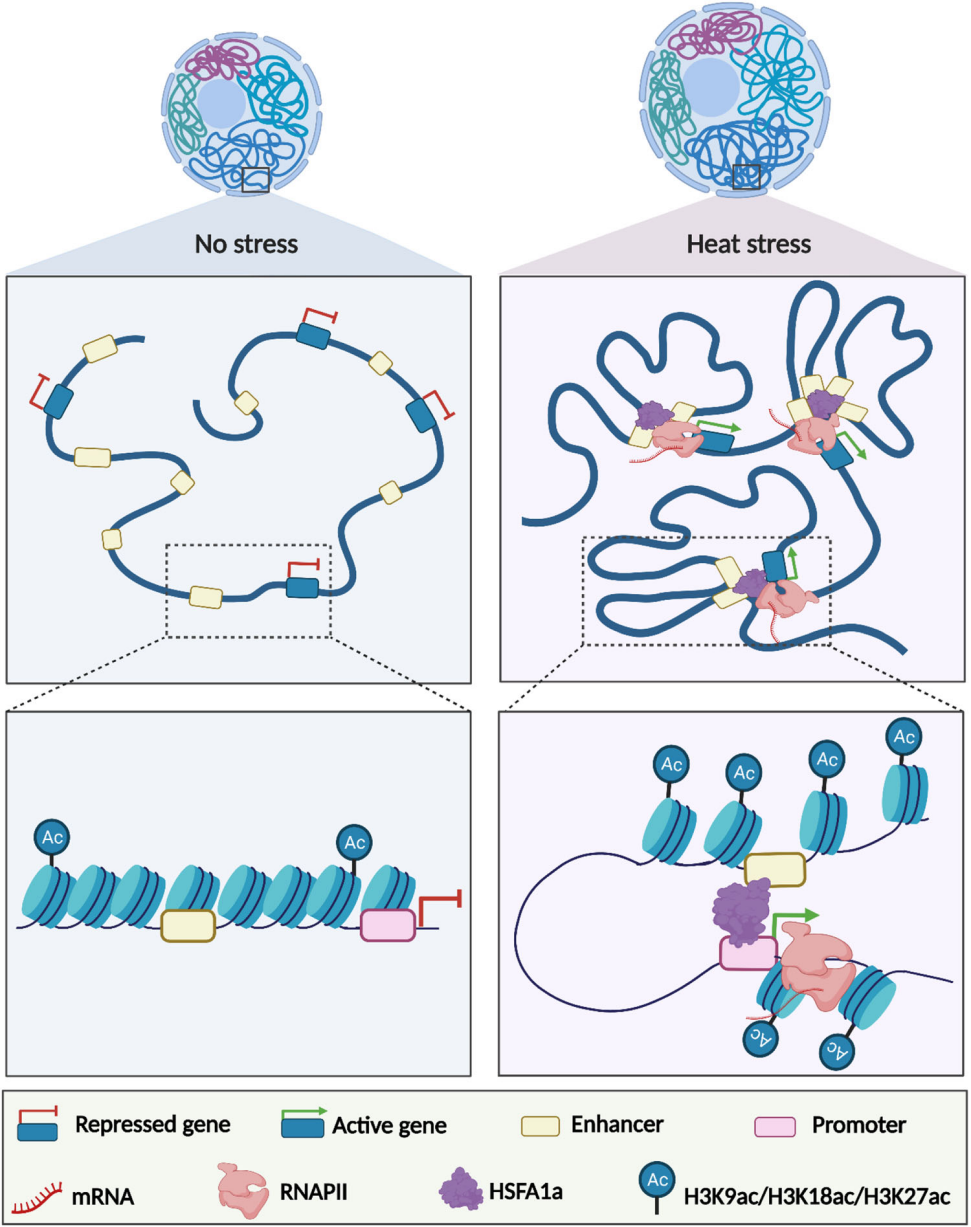
Model recapitulating the role of HSFA1a in enhancer-promoter loop formation to regulate gene expression in response to heat stress in tomato. The diagram was created using BioRender.

To assess whether HSFA1a influences RE-promoter loop formation, we focused our analysis on *Solyc09g074475* and *HSFA2*. These two genes were selected because they are both heat-responsive in an HSFA1-dependent manner, they display an HSFA1a binding site in their promoter region, and dynamic RE-promoter contacts in response to HS. Furthermore, HSFA2 is well-known as a key component of thermotolerance in *Arabidopsis*^41^. C-Hi-C and HiChIP-RNAPII revealed the presence of a discrete RE that interacts with *Solyc09g074475* and an unannotated gene, and that this interaction was enhanced in response to HS (Fig. 5e, Supplementary Fig. 25a). Notably, the formation of these two chromatin loops correlated with a strong activation of both genes in response to HS, thus suggesting that this element may act as a transcriptional enhancer. To test whether this putative RE has the potential to function as an enhancer, we generated a construct containing this RE sequence fused to a minimal mosaic virus (CaMV) 35 S promoter to drive the expression of a luciferase (LUC) reporter after transformation of tobacco -*N. benthamiana* leaves. We found that compared to the minimal promoter, the addition of the *Solyc09g074475* RE sequence significantly increased the expression of the LUC reporter (*P* = 8.40e-05, Student’s *t*-test) (Fig. 5e). These results supported the view that this genomic region can act as a transcriptional enhancer. To assess whether HSFA1a is necessary for the formation of chromatin loops with this regulatory sequence, we used chromosome conformation capture (3 C) followed by qPCR (3C-qPCR) on plants grown under control conditions or subjected to 1 h of HS. We found that in WT plants, the strength of the two loops, named A and B, increased after 1 h of HS, whereas in *hsfa1a* knock-down transgenic lines the strength of the two loops did not increase but instead was significantly reduced (Loop A: *P1* = 3.20e-05*, P2* = 3.20e-05 and Loop B: *P1* = 8.15e-05*, P2* = 4.23e-05, Student’s *t*-test) (Fig. 5f). With respect to the *HSFA2* locus, C-Hi-C and HiChIP -RNAPII revealed that it is potentially regulated by a chromatin loop with two distal REs (named distal RE-A and distal RE-B) (Fig. 5g, Supplementary Fig. 25b). The formation of this chromatin loop correlated with the activation of *HSFA2*, thus suggesting that these elements could act as transcriptional enhancers. To test this, we followed the previously described approach and found that compared to the control minimal promoter construct, the signal strength of either distal REs was significantly increased (RE-A, *P* = 3.21e-04; RE-B, *P* = 1.87e-04) (Fig. 5g). We used 3C-qPCR to determine whether HSFA1a influences this distal RE-promoter loop formation, and found that depleting HSFA1a decreased the strength of HS-specific contacts between the distal putative enhancer and the *HSFA2* locus (*P1* = 3.26e-08*, P2* = 3.26e-08, Student’s *t*-test) (Fig. 5h). Similar experiments were conducted with two additional candidate enhancers interacting with genes involved in heat stress response, leading to the same results (Supplementary Fig. 26). Collectively, our results suggest that HSFA1a plays a major role in the dynamic formation and/or stability of promoter-enhancer interactions that underpin the initial transcriptional responses to HS (Fig. 6).

## Discussion

This study presents a deep analysis of the tomato nuclear architecture by integrating capture-Hi-C, ATAC-seq, ChIP-seq, HiChIP, and RNA-seq data. Results obtained highlight the specific functionalities of the different levels of chromatin spatial organization underscoring the regulatory importance of nuclear organization as a whole. Chromatin conformation in tomato is strongly compartmentalized and organized around focalized sites of transcription in the active chromatin that resemble transcription factories^3,42^. Although the precise mechanisms underlying transcription factories organization remain poorly understood, they are thought to play a critical role in clustering co-regulated genes^43^. The presence of transcription factories in the tomato nucleus reveals their importance in transcriptional regulation and in particular on the optimization of transcription in response to environmental cues by concentrating TFs in discrete nuclear compartments.

Temporal and spatial gene expression is influenced by dynamics chromatin changes and is implicated in the control of developmental programs as well as in response to environmental stresses^14,21^. In metazoan interphase nuclei, chromatin is organized into TADs, which are thought to bring together co-regulated genes. In Drosophila, HS induces dramatic rearrangement of the 3D nuclear organization^44^, suggesting that TAD organization of metazoan genomes is plastic and is reconfigured in response to environmental changes^25^. However, a recent study in human and Drosophila cells has shown that HS only affects a subset of enhancer–promoter interactions whereas TADs and compartment structures remain mostly stable upon HS^45^. In plants, HS induces high-order reorganization of the constitutive heterochromatin compartment^22,28^. In line with these results, we observed that HS in tomato negatively affects the chromatin interactions between constitutive heterochromatin regions, thus affecting the strength of constitutive heterochromatin compartmentalization.

REs are non-coding DNA sequences containing binding motifs for one or several TFs involved in transcriptional regulation of target genes, and are also key components of the 3D chromatin organization. These sequences act as proximal or distal RE and their activity is reflected by their accessibility, which is likely controlled by the action of pioneer TFs and chromatin modifiers^46,47^. In animals, distal REs are associated with the specific histone marks H3K27ac and H3K4me1, defining a specific chromatin state^48^. By contrast, in plants, studies in maize and wheat have proposed that H3K27ac, H3K4me3 and H3K9ac are histone marks preferentially associated with distal REs^49–51^. Here, we focused on differentially accessible REs as a proxy for active REs and found that active distal and proximal REs displayed different chromatin signatures. We have found that in tomato distal REs are characterized by the presence of nucleosomes rich in H3K9ac and H3K18ac and devoid of H3K4me3, flnaking the region with maximal accessibility. Importantly, we demonstrated that some of these RE are capable to act as transcriptional enhancers.

3D chromatin architecture dynamically controls the access of REs to their target genes by promoting or inhibiting RE-promoter interactions. RE sequences can be hundreds to thousands of base pairs upstream or downstream from the gene they regulate, making it difficult to define which REs control which genes. The rapid development sequencing technologies has enabled multiple high-throughput experimental methods to measure chromatin interactions, including Capture-Hi-C and HiChIP, which are particularly useful to detect enhancer–promoter interactions. In animals, promoter C-Hi-C has been used for the analysis of long-range RE-promoter interactions and their control of gene expression using different cell types. These analyses have revealed a massive change in RE-promoter interactions taking place during cellular differentiation^52–56^. In plants, RE-promoter interactions were analyzed in maize using HiChIP for H3K4me3, H3K27me3 and H3K27ac, which identified long range RE-promoter interactions that contribute to the control of gene expression^57,58^. This analysis uncovered the existence of long-range RE-promoter interactions. In line with this observation, using both capture-Hi-C and HiChIP approaches in tomato, several thousands of RE-promoter interactions were discovered. Notably, we have found examples for a promoter interacting with several REs as well as examples where one RE interacts with multiple genes. These two types of chromatin could be defined as promoter-centric hubs or RE-centric hubs respectively^59^.

One of the mechanisms proposed to shape the 3D genome structure in animals is the loop extrusion model, that involves the structural maintenance of chromosomes (SMC) cohesin complex and the zinc-finger DNA-binding protein CTCF^60,61^. Plants genomes appear to lack CTCF homologues, but SMC complexes are highly conserved. Therefore, this loop extrusion may be conserved in plants and involve yet unknown factors that act as insulators. In addition to the action of SMC and CTCF, TFs serve as protein anchors and determine 3D genome organization^35^. TFs may mediate RE-promoter interactions through different mechanisms, including direct protein homo-dimerization^62^ and co-factor protein recruitment to generate oligomers^35^. In *Arabidopsis*, the CONSTANS TF plays a crucial role in forming of an enhancer-promoter loop to control the expression of *FLOWERING LOCUS T*^63^, thus suggesting that TFs also impact 3D chromatin organization in plants. However, most of studies in plants have focused on deciphering the role of TFs in 3D chromatin architecture and gene regulation under steady-state conditions, thus providing clues on the mechanisms involved in the maintenance of RE-promoter interaction rather than in their formation. To decipher the molecular mechanisms involved in establishing distal RE-promoter interactions, we carried out a multidimensional study combining stress at different time points. Our data show that 3D chromatin interactions are dynamically modified by HS to allow a temporally evolving connection between a diversity of activated REs and promoters. Furthermore, our integrative analyses revealed that the binding motif of HSF was over-represented in the sequences involved in RE–promoter contacts at the onset of HS treatment. These data suggest that transcription factors of the HFS family play a major role in RE–promoter contact formation. Studies in yeast have shown that HSF1 mediates the clustering of its targets and dynamically influences the reconfiguration of the genome^35,64^. Our data for tomato indicates that HSFA1a plays a critical role in the dynamic formation of promoter-enhancer contacts and in controlling the transcriptional response at the onset of HS. Future work shall help determine whether loop formation is a pre-requisite for transcription activation, or is rather a consequence of it. Few studies have assessed the dynamic role of TFs in the formation of chromatin loops in response to a stimulus, even in animals. One study revealed that KLF4 and ZNF750 TFs, rather than cohesins, are key to establishing of RE-promoter contacts to promote gene expression during epidermal cell differentiation^55^. Collectively, our findings shed light on the complexity of distal and proximal RE contact loop formation and provides evidence for a key role of TFs in controlling transcriptional response through the 3D reconfiguration of the chromatin structure in animals and plants.

## Methods

### Plant material and growth conditions

Tomato (*Solanum lycopersicum*) lines used in this study were in the cv. M82 and cv. Moneymaker backgrounds. The *hsfa1a* mutants line1 and line2 were obtained from Dr.Fragkostefanakis’s lab^27^. Seeds were directly sown on soil and plants grown in growth chambers at 24 °C under long-day (16 h light) conditions. For heat treatment, 4-week-old plants were treated at 45 °C for 1 h and 6 h in a climatic chamber (Aralab). The fourth leaf was used for all experiments in this research.

### Immunostaining

Immunostaining experiments were performed according to Latrasse et al^65^. Briefly, fourth leaves of 4-week-old tomato plants were fixed and then nuclei were isolated, placed on a poly-lysine slide, and incubated overnight at 4 °C with primary antibodies (400X diluted) of H3K9me2 (Abcam, ab1220), H3K9ac (Millipore, 07–352), H3K27me1 (Millipore, 07–448) and RNAPII (Abcam, ab26721). Slides were washed and then incubated for 1 h at room temperature in the dark with Goat anti-Rabbit Alexa Fluor Plus 488 (A11034 Invitrogen) and Goat anti-Mouse Alexa Fluor Plus 555 (A32727 Invitrogen) or with Goat anti-Rabbit Alexa Fluor Plus 555 (A32732 Invitrogen) and Goat anti-Mouse Alexa Fluor Plus 488 (A32723 Invitrogen) secondary antibodies (400X diluted). DNA was counterstained with 4,6 diamidino-2-phenylindole (DAPI) in SlowFade Diamond Antifade mounting media. Slides were directly imaged on a confocal microscope (Zeiss Microsystems).

### Assay for transposase-accessible chromatin with high-throughput sequencing

Approximately 100 mg of 4-weeks-old tomato fourth leaves were ground, passed through a 100 µm filter and the nuclei were isolated using nuclei isolation buffer (0.25 M sucrose, 10 mM Tris-HCl pH8, 10 mM MgCl2, 1% Triton X-100, 5 mM 2-mercapto-ethanol, and 0.1 mM protease inhibitors). The nuclei were re-suspended in 2× TD buffer (20 mM Tris-HCl, pH 7.6, 10 mM MgCl2, and 20% dimethyl formamide) and 2.5 µl of Tn5 transposase (Illumina FC-121-1030). The transposition reaction was performed at 37 °C for 30 min, and DNA was purified using a Qiagen MinElute Kit (QIAGEN, Cat.No.28004). DNA libraries were amplified for a total of 10 cycles as described by Buenrostro et al^66^ and Jegu et al^67^. DNA libraries were checked for quality and quantified using a 2100 Bioanalyzer (Agilent) and subjected to 2 × 75 bp high-throughput sequencing by NextSeq 500 (Illumina). Two independent biological replicates were generated.

### RNA-seq assay

Total RNA was extracted from the fourth leaves of 4-week-old tomato with Nucleospin RNA kit (Macherey-Nagel), according to the manufacturer’s instructions. RNA-seq libraries were prepared from 2 μg of total RNA using NEBNext Ultra II Directional RNA library Preparation Kit (NEB) according to the manufacturer’s instructions. RNA libraries were checked for quality and quantified using a 2100 Bioanalyzer (Agilent) and subjected to 1 × 75 bp high-throughput sequencing by NextSeq 500 (Illumina). Two independent biological replicates were generated.

### In situ Hi-C assay

In situ Hi-C experiments were performed according to Concia et al.^3^ using DpnII enzyme (New England Biolabs). DNA libraries were prepared using NEBNext Ultra II DNA library preparation kit (NEB) according to the manufacturer’s instructions (10 cycles for the PCR amplification step). DNA libraries were checked for quality and quantified using a 2100 Bioanalyzer (Agilent) and the libraries were subjected to 2 × 75 bp high-throughput sequencing by NextSeq 500 (Illumina). Two independent biological replicates were generated.

### C-Hi-C assay

For C-Hi-C, the in situ Hi-C libraries were used for the capture step. Capture was performed by using SureSelect XT Target Enrichment System for Illumina Paired-End Multiplexed Sequencing Library (Agilent) according to the manufacturer’s recommendations. All tomato cultivar M82 promoters were selected1.5 kb in length and 212,735 probes (length of each probe = 120 bp) were designed to catch all promoters. The capture step was performed using 1 µg of in situ Hi-C libraries following the manufacturer’s recommendations. The quality of the libraries was assessed using a 2100 Bioanalyzer (Agilent), and the libraries were subjected to 2 × 75 bp paired-end high-throughput sequencing by NextSeq 500 (Illumina). Two independent biological replicates were generated

### HiChIP assay

Nuclei were isolated from the fourth leaves of 4-week-old tomato plants using the same procedure as for the in situ Hi-C experiments. The Hi-ChIP protocol from Mumbach et al.^68^ was then applied using the DpnII restriction enzyme (NEB) and 3ug of anti-RNAPII antibody (Abcam, ab26721). The quality of the libraries was assessed using a Bioanalyzer (Agilent), and the libraries were subjected to 2 × 75 bp paired-end high-throughput sequencing by NextSeq 500 (Illumina).

### ChIP-seq assay

ChIP-seq assays were performed on the fourth leaves of 4-week-old tomato plantsaccording to Bio-protocol of Ramirez-Prado et al.^69^ using 3ug of anti-H3K27me1 (Millipore, 07–448), anti-H3K9ac (Millipore, 07–352), anti-H3K14ac (Millipore, 07–353), anti-H3K18ac (Millipore, 07–354), anti-H3K27ac (Abcam, ab4729), anti-H3K4me3 (Millipore, 07–473), and anti-RNAPII (Abcam, ab26721) antibodies. ChIP-seq libraries were prepared from 10 ng of DNA using NEBNext Ultra II DNA Library Prep Kit for Illumina (NEB) according to the manufacturer’s instructions. Two independent biological replicates were generated for each time point of heat stress. DNA libraries were checked for quality and quantified using a 2100 Bioanalyzer (Agilent) and subjected to 1 × 75 bp high-throughput sequencing by NextSeq 500 (Illumina).

### Amplified DNA affinity purification sequencing assay (AmpDAP-seq)

AmpDAP-seq assays were performed according to Bartlett et al.^40^ with modifications. Briefly, gDNA was extracted from 4-week-old tomato fourth leaves, fragmented, and a library was generated using NEBNext Ultra II DNA library preparation kit (NEB, #E7645S/L, #E7103S/L). *SolycHsfA1a* open reading frames were transferred into the Gateway-compatible pIX-HALO expression vector and expressed using Promega TNT Coupled Wheat Germ Extract Systems (Promega, cat. no. L4130). The expressed proteins were immobilized on Magne HALO-Tag beads (Promega, G728A), washed, and incubated with the DNA library. After bead washing, DNA was eluted and amplified with indexed NEBNext primers (20 PCR cycles were used). DNA libraries were checked for quality and quantified using a 2100 Bioanalyzer (Agilent) and subjected to 1 × 75 bp high-throughput sequencing by NextSeq 500 (Illumina). Two independent biological replicates were generated.

### 3C-qPCR

Four-week-old tomato fourth leaves were cross-linked in 1% (v/v) formaldehyde at room temperature for 20 min. Cross-linked plant material was ground and nuclei were isolated and treated with 0.5% SDS at 62 °C for 5 min. The SDS was then supplemented with 2% Triton X-100. Digestions were performed overnight at 37 °C using 150U of DpnII enzyme (New England Biolabs). Restriction enzymes were inactivated by adding 1.6% SDS and incubated at 62 °C for 20 min. SDS was supplemented with 1% Triton X-100. DNA was ligated by incubating at 22 °C for 5 h using 4000U of T4 DNA ligase (Fermentas). Reverse crosslinking was performed by overnight treatment at 65 °C. DNA was recovered after Proteinase K treatment by phenol-chloroform extraction and ethanol precipitation. Relative interaction frequencies were calculated by quantitative PCR using 15 ng of DNA. A region uncut by DpnII was used to normalize the amount of DNA. Details for primers used for 3C-qPCR are listed in Supplementary Data 3. Two independent biological replicates were generated.

### Dual-luciferase assay

Luciferase activity was quantified using reagents from the Dual Luciferase Reporter Assay System (Promega, E2920) according to the manufacturer’s instructions.

### Analysis of ChIP-seq data

Trimmomatic-0.38^70^ was used for trimming with the following parameters: minimum length of 36 bp; mean Phred quality score >30; leading and trailing bases removal with base quality <5. The reads were mapped onto the M82 V1.0^71^ assembly using Bowtie2 V2.3.5^72^ with mismatch permission of 1 bp (Supplementary Data 4). To identify significantly enriched regions, we used MACS2 V2.2.7.1^73^ with the following peak-calling parameters: number of duplicate reads at a location: 1; mfold of 5:50; q-value cutoff: 0.05; extsize 200; broad peak. To extract the average scores across the genomic regions, multiBigwigSummary command from the deepTools package^74^ was used with default parameters on the RPGC normalized bigWig files.

### Differential expression analysis

Single-end reads of RNA-seq samples were trimmed using Trimmomatic-0.38 with the parameters: minimum length of 30 bp; mean Phred quality score >30; leading and trailing base removal with base quality <5. STAR aligner^75^ was used to map the reads to the M82 V1.0^71^ genome assembly (Supplementary Data 4). Raw read counts were then extracted using the featureCounts V2.0.0 utility from the R Subread package based on the M82_v1.1.0 gene annotations^71^. Finally, we used DESeq2 V1.38.0^76^ to identify differentially expressed genes. Genes with read counts ≥ 50 in at least 2 samples were considered for differential expression analysis.

### Analysis of HiChIP and C-Hi-C data

Raw FASTQ files were preprocessed with Trimmomatic-0.38 to remove Illumina sequencing adaptors. The 5′ and 3′ ends with a quality score below 5 (Phred+33) were trimmed and reads <30 bp after trimming were dropped. The trimmed files were then processed with HiC-Pro V2.11.4^77^. The reads were aligned using Bowtie2 V2.3.5 onto the M82V1.0 assembly with default settings, except for the parameter “–score-min L, −0.6, −0.8” (Supplementary Data 4). Invalid ligation products (such as dangling ends, fragments ligated on themselves, and ligations of juxtaposed fragments) were discarded. Valid pairs were used to produce raw interaction matrixes at various resolutions. Finally, “.hic” files were generated with the software Juicer Tools and visualized with the tool Juicebox^78^.

### Identification and analysis of genomic interactions

Valid pairs generated were further analyzed using HOMER V4.11^79^ for different resolutions (500 bp for short-range interactions and 20 kb for long-range interactions). For the short range interactions, both anchors of the interactions (genomic bins) were annotated with genes using bedtools intersect^80^. We removed interactions without any gene annotations, self-loops, and duplicates.

### Reporting summary

Further information on research design is available in the Nature Portfolio Reporting Summary linked to this article.

## Supplementary information

Supplementary Information

Description of additional Supplementary File

Supplementary Data 1

Supplementary Data 2

Supplementary Data 3

Supplementary Data 4

Reporting Summary

## Data Availability

The data that support this study are available from the corresponding author upon reasonable request. Raw sequencing data generated in the course of this study have been deposited to the Gene Expression Omnibus (GEO) database under accession number GSE206365. Source data are provided with this paper.

## Source data

Source data
