# Supplementary material for: HSFA1a modulates plant heat stress responses and alters the 3D chromatin organization of enhancer-promoter interactions
Source: Nat Commun. 2023 Jan 28;14:469. doi: 10.1038/s41467-023-36227-3 (PMC9884265; doi:10.1038/s41467-023-36227-3)
Supplement: Supplementary file 1 — Supplementary Information [file 41467_2023_36227_MOESM1_ESM.pdf]

a

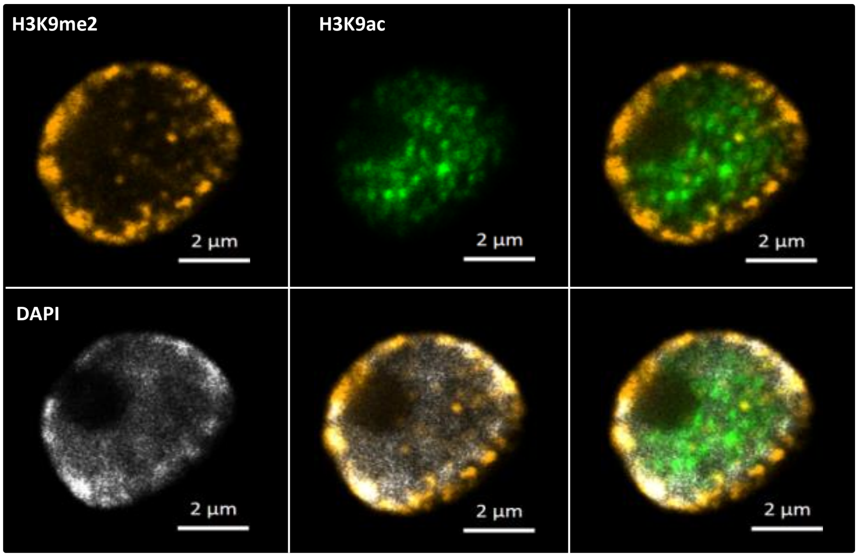

b

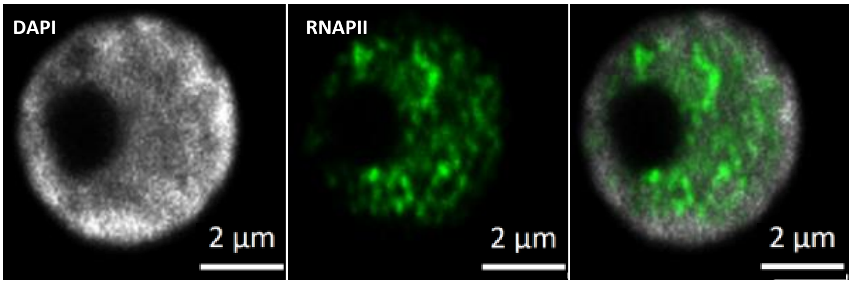

Supplementary Figure 1. Chromatin organization in tomato
(a) Immunofluorescence detection of H3K9me2 (orange), H3K9ac (green), RNAPII (green), and DAPI staining (grey) in an isolated tomato nucleus. (b) Immunofluorescence detection of RNAPII (green) and DAPI staining (grey) in an isolated tomato nucleus. 3 times each experiment was repeated independently with similar results.

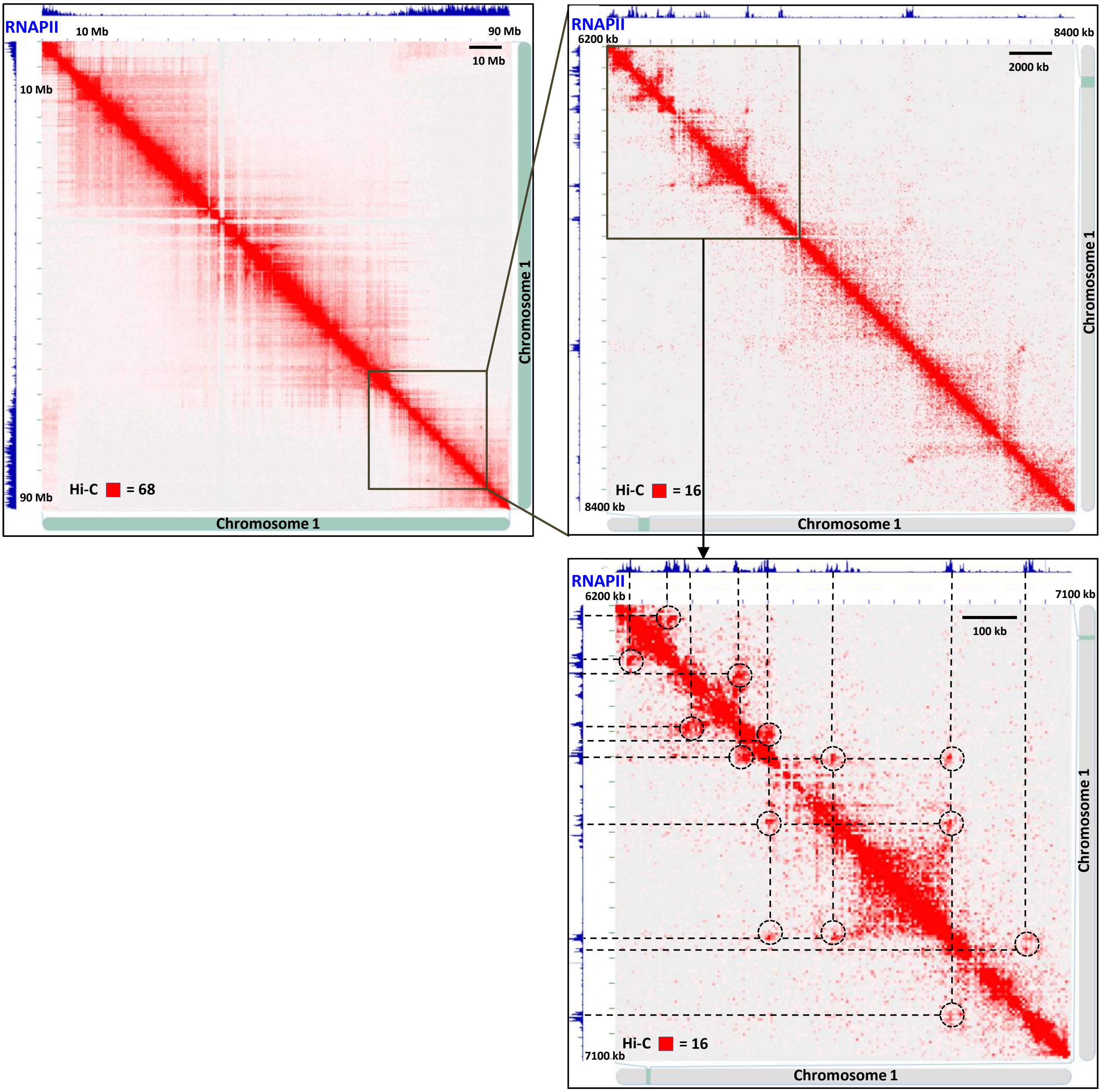

Supplementary Figure 2. Tomato chromatin is organized around transcription factories
Visualization of the Hi-C interaction matrix in a specific region of the chromosome 1. RNAPII ChIP-seq signal (blue peaks) was aligned with the maps to highlight the loops.

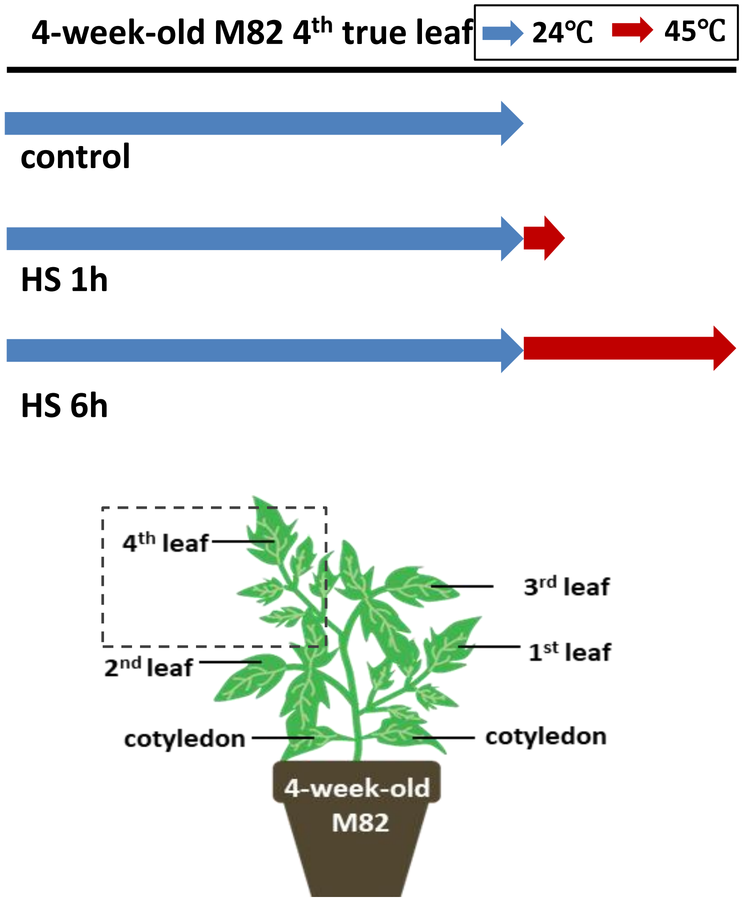

Supplementary Figure 3. Heat stress protocol

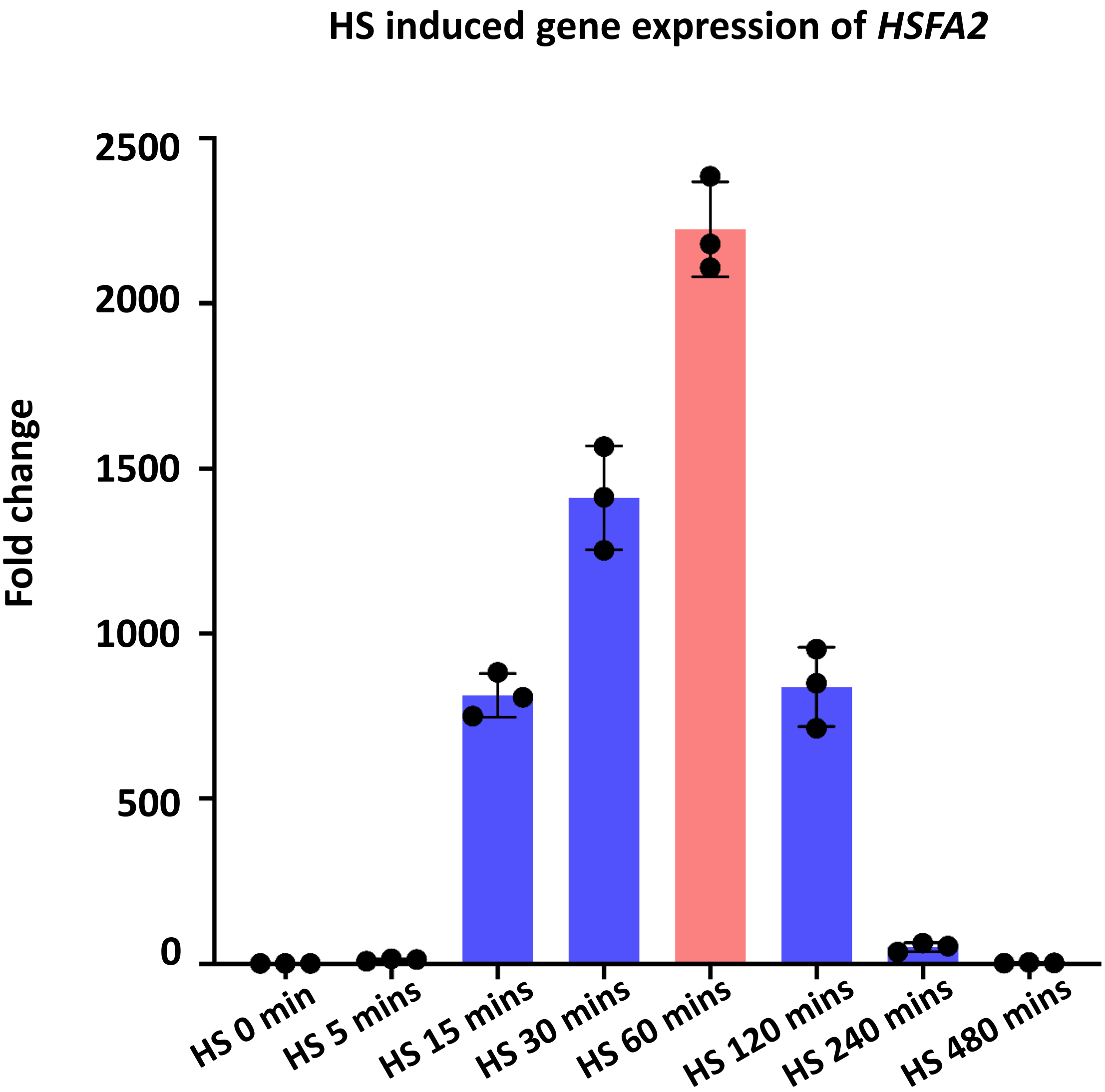

Supplementary Figure 4. Gene expression pattern of *HSFA2* after heat stress treatment at different time points (0, 5, 15, 30, 60, 120, 240,480 mins) in wild type. Data are presented as mean values +/- standard deviation (SD) from three biological replicates (n=3). Source data are provided as a Source Data file.

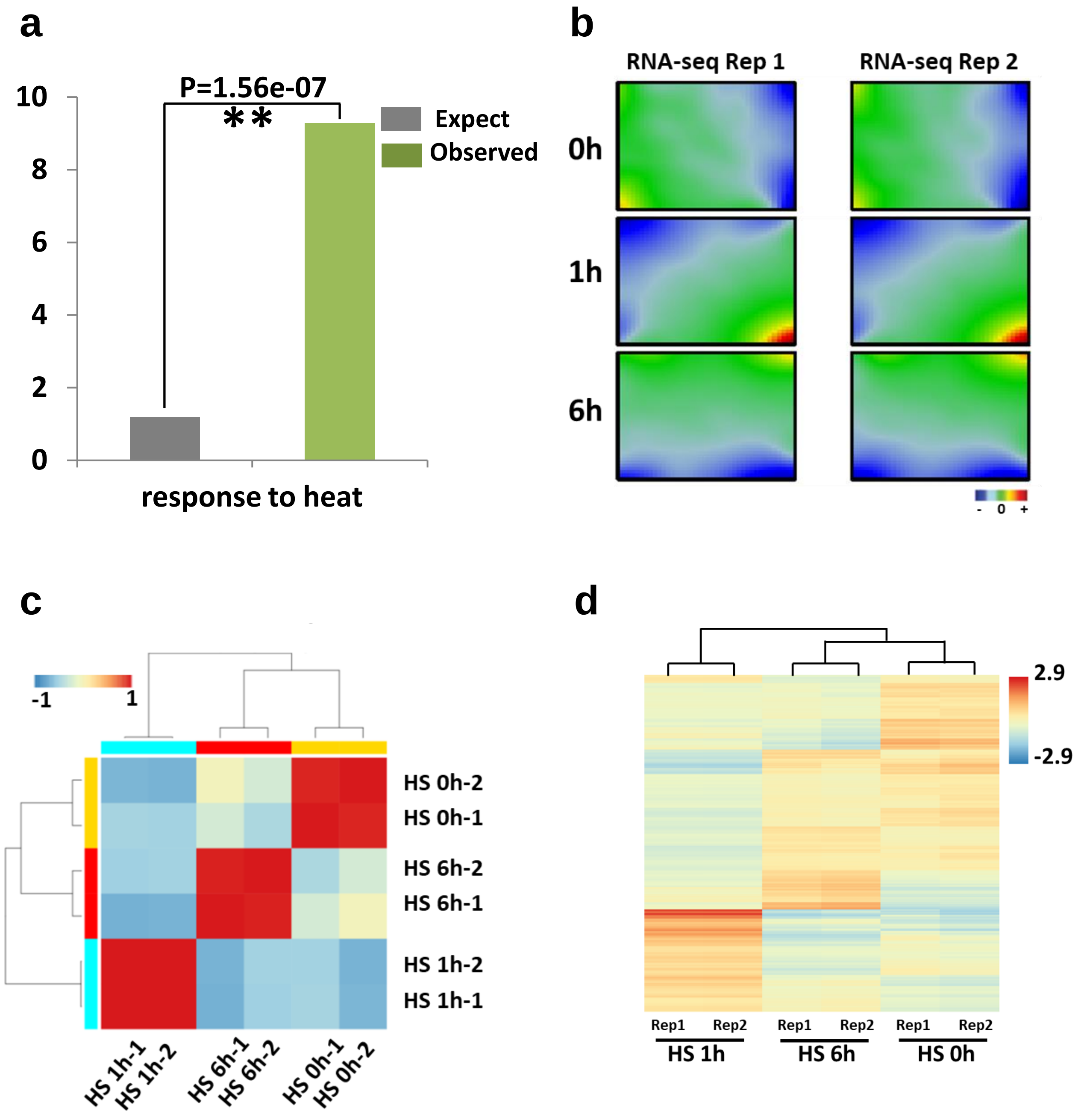

Supplementary Figure 5. Heat stress-induced transcriptome reprogramming in tomato
(a) Gene ontology analysis of upregulated genes after 1 h of heat stress (n=1247). P values calculated using Fisher's exact test. (b) Gene expression landscapes generated with the opoSSOM R package after 0, 1 and 6 h of heat stress. (c) Correlation of the gene expression datasets after 0, 1 and 6 h of heat stress. (d) Heatmap of the gene expression level after 0, 1 and 6 h of heat stress. Source data are provided as a Source Data file.

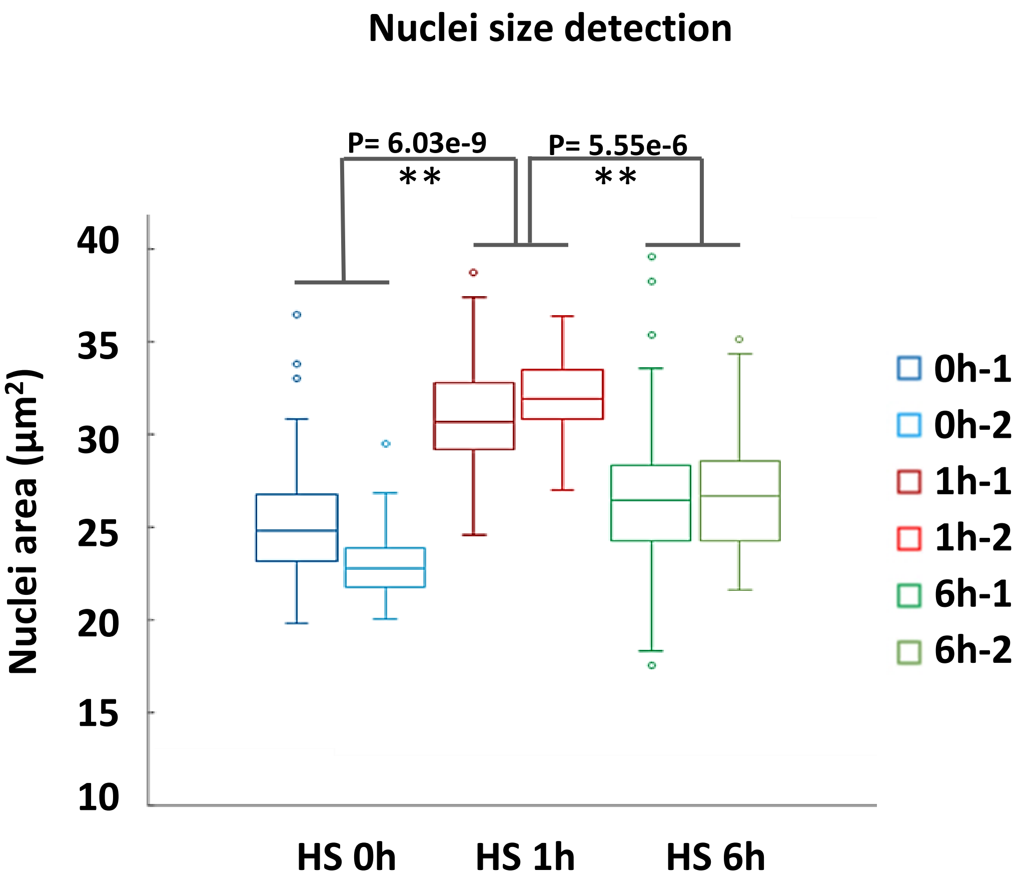

Supplementary Figure 6. Nuclei size measurement after 0, 1, or 6 h of heat stress Nuclei were stained with DAPI and nuclei area was analyzed after 0, 1, or 6 h of heat stress by using ImageJ. 2 biologically independently replicates were detected (n1-HS 0h-1=5195; n2-HS 0h-2=4626; n3-HS 1h-1=10515; n4-HS 1h-2=12006; n5-HS 6h-1=10987; n6-HS 6h-2=14094). P-value was determined by the two-sided Mann–Whitney U test. For each box plot, center lines indicate the medians; boxes show the 25th and 75th percentiles; whiskers extend to the minimum and maximum. Source data of Supplementary Figure 6 are provided as a Source Data file.

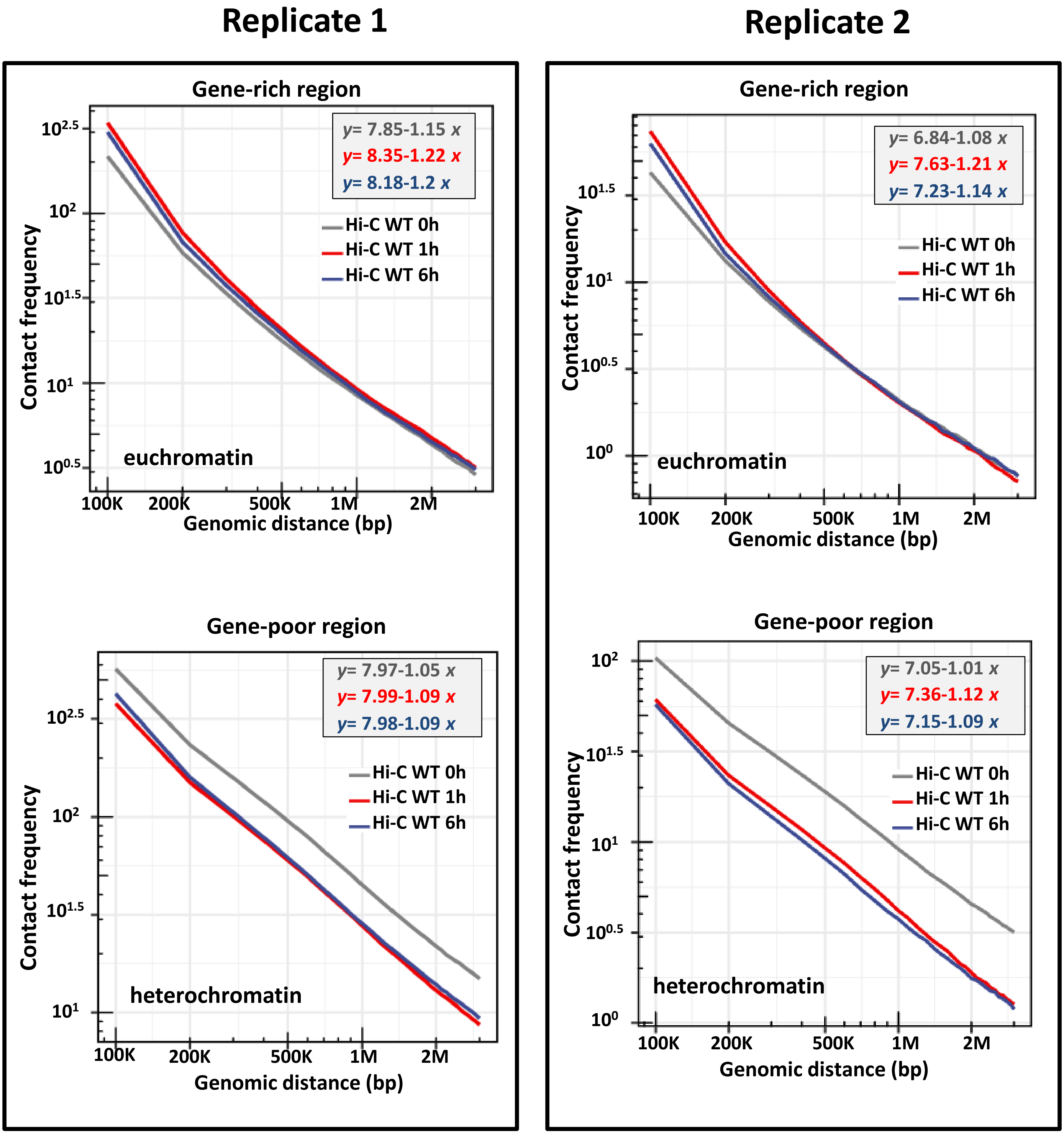

Supplementary Figure 7. Replicated averaged scaling plots of interaction frequencies against increasing genomic distance. The genomic bin size is 100 kb.

Chromatin compartmentalization

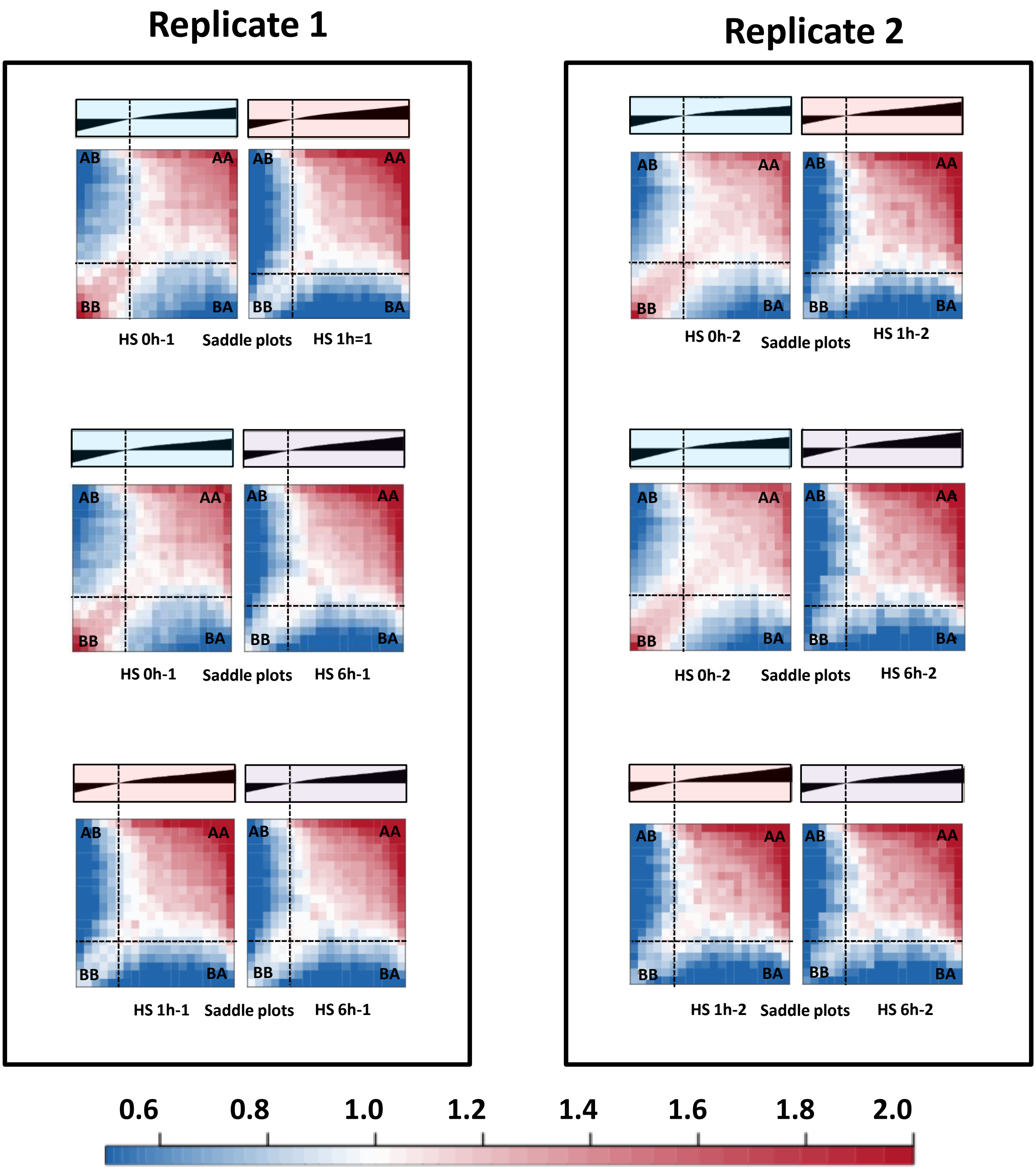

Supplementary Figure 8. Heat impacts chromatin compartmentalization
Saddle plots of chromatin compartmentalization.

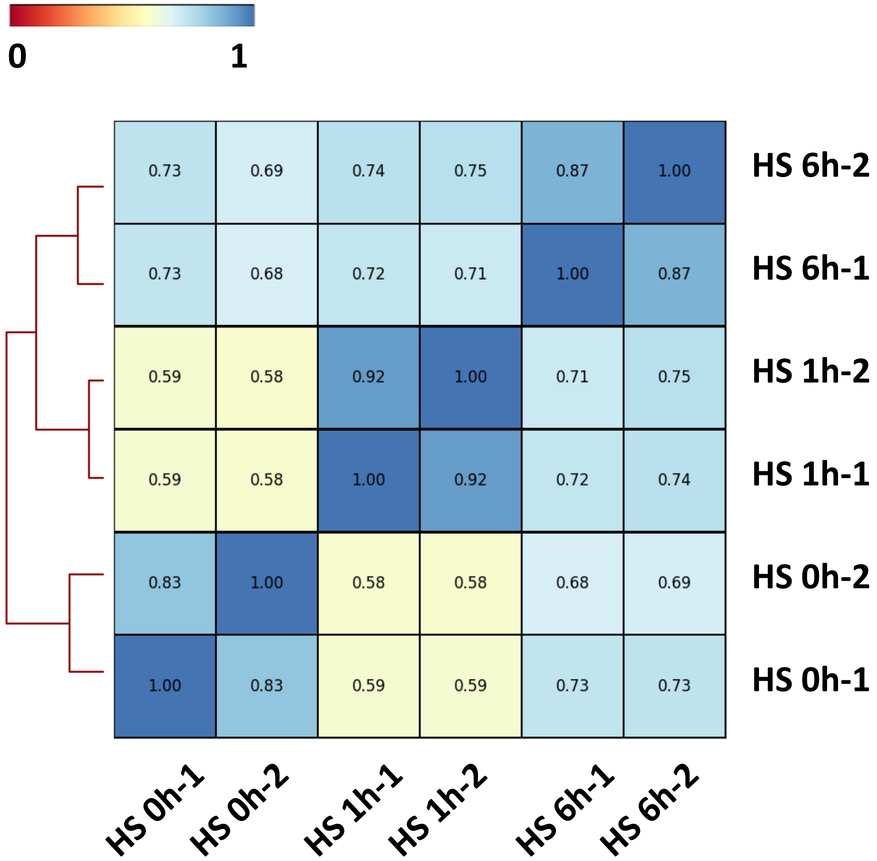

Supplementary Figure 9. Correlation of the DNA accessibility between replicates after 0, 1, and 6 h of HS. Color scale shows the correlation level.

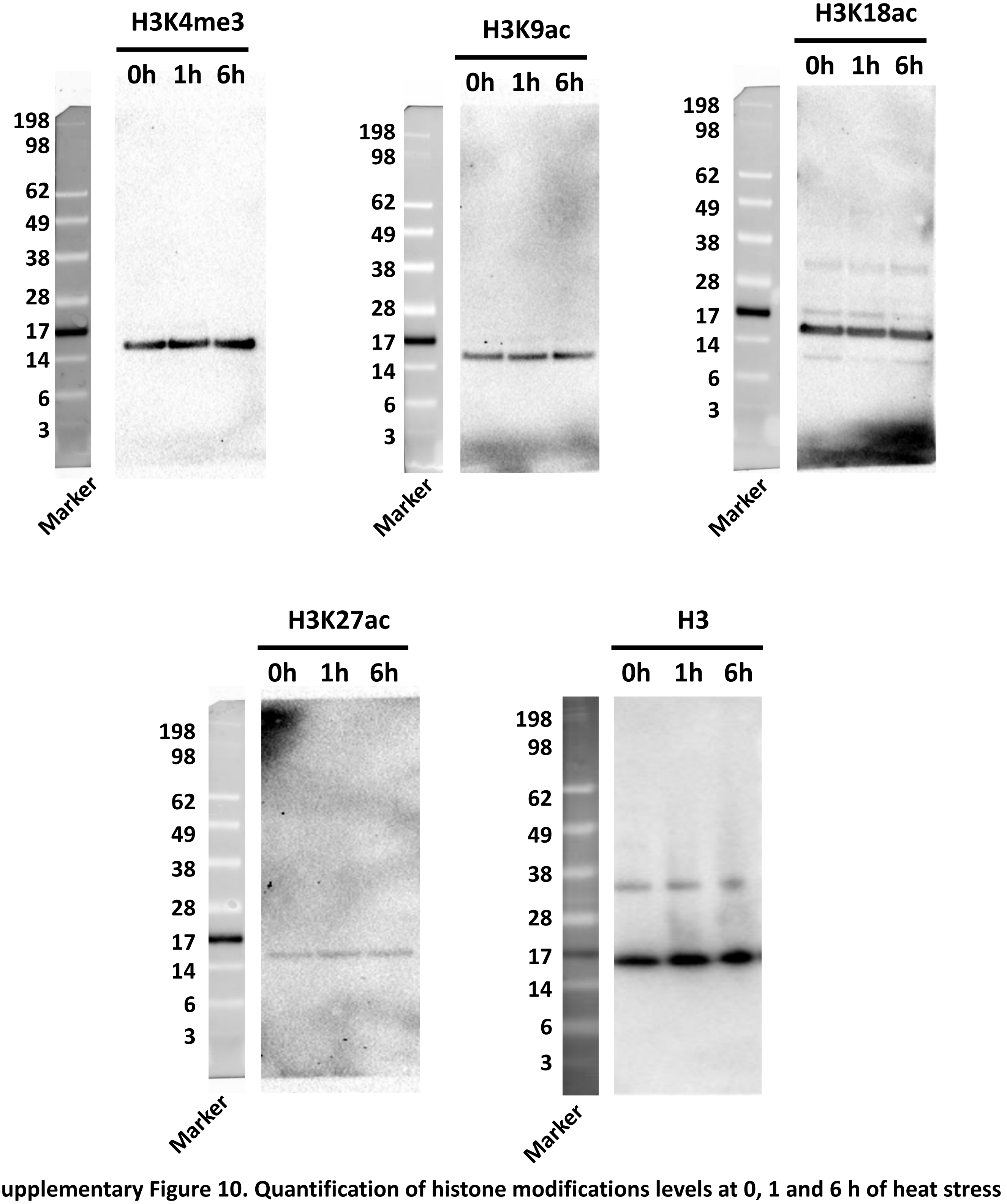

Supplementary Figure 10. Quantification of histone modifications levels at 0, 1 and 6 h of heat stress H3K4me3, H3K9ac, H3K18ac, H3K27ac and H3 levels were monitored by immunoblotting at 0, 1, and 6 h of heat stress. The molecular weight marker is shown on the left. 3 times each experiment was repeated independently with similar results. Source data are provided as a Source Data file.

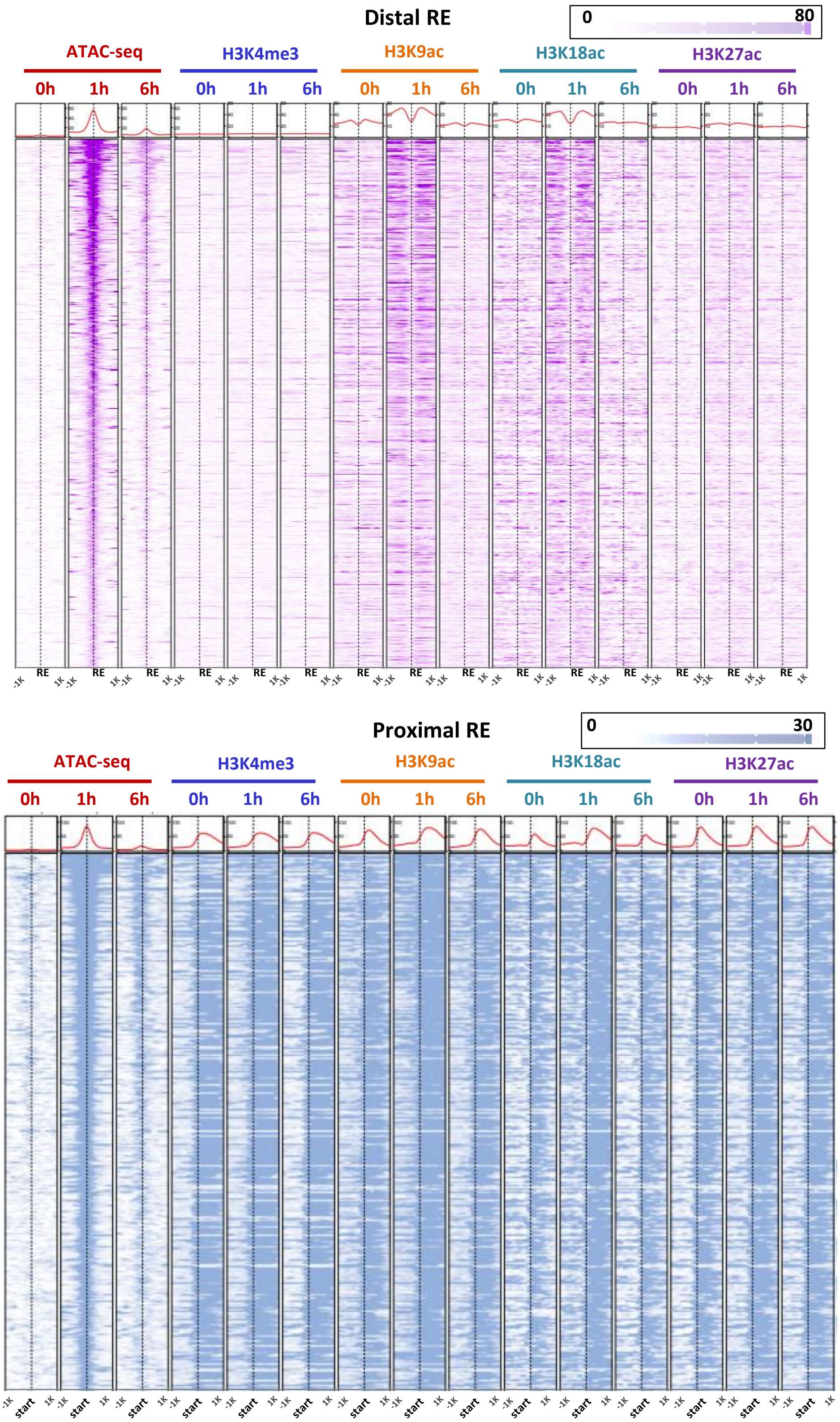

Supplementary Figure 11. Heatmaps of ATAC-seq and ChIP-seq (H3K4me3, H3K9ac, H3K18ac and H3K27ac) profiles over proximal (grey) and distal (purple) regions at 0, 1 and 6 h of heat stress.

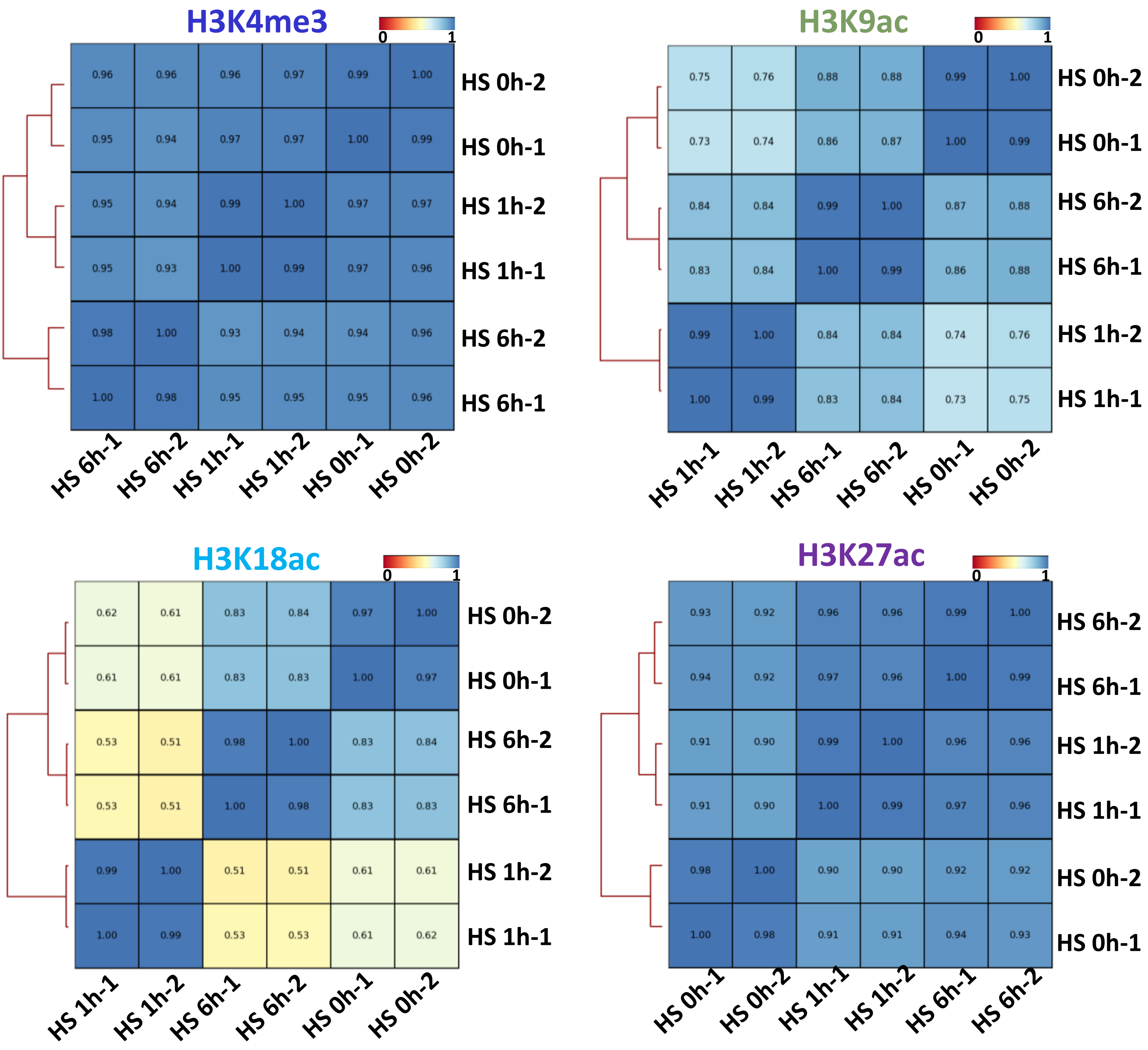

Supplementary Figure 12. Correlation of the ChIP-seq between replicates after 0, 1, and 6 h of HS.

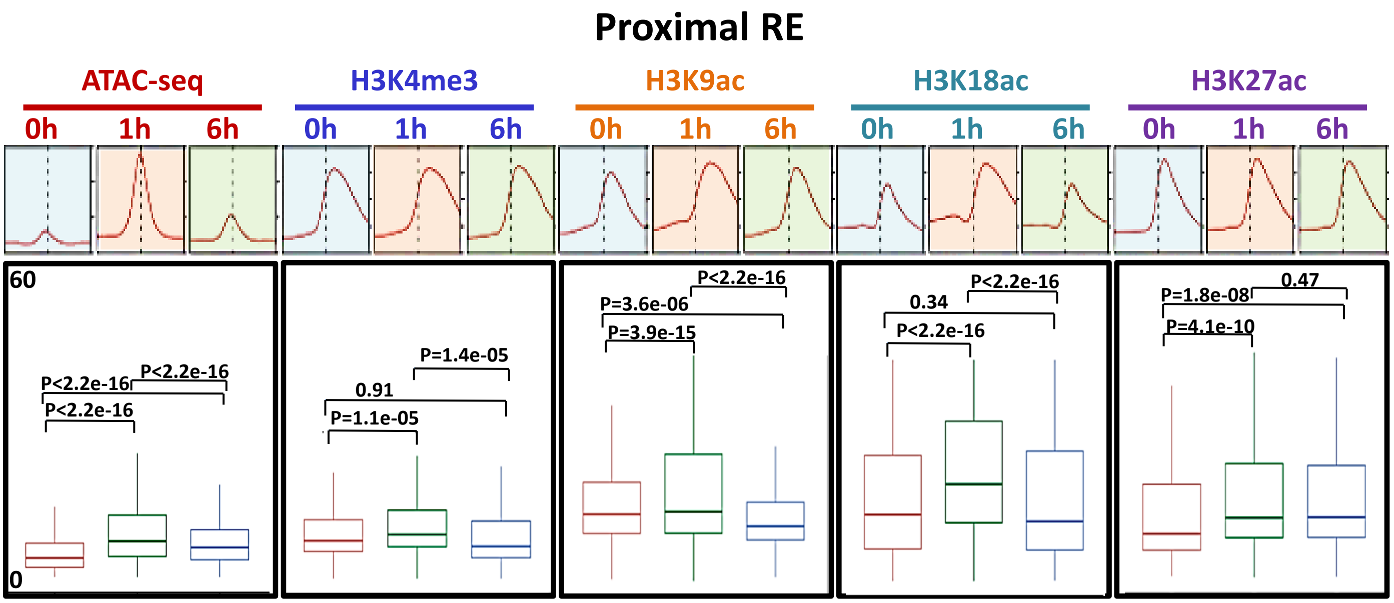

Supplementary Figure 13. Histone marks enrichment of H3K4me3, H3K9ac, H3K18ac, H3K27ac after 0, 1, and 6 h heat stress treatments in proximal regulatory elements. Each enrichment score was calculated as the corresponding S3norm normalized signal of proximal regions. Each feature was compared with t test (n=3778) along with multiple comparisons and adjusted P values. For each box plot, center lines indicated the medians; boxes showed the 25th and 75th percentiles; outlier values have been removed. Source data are provided as a Source Data file.

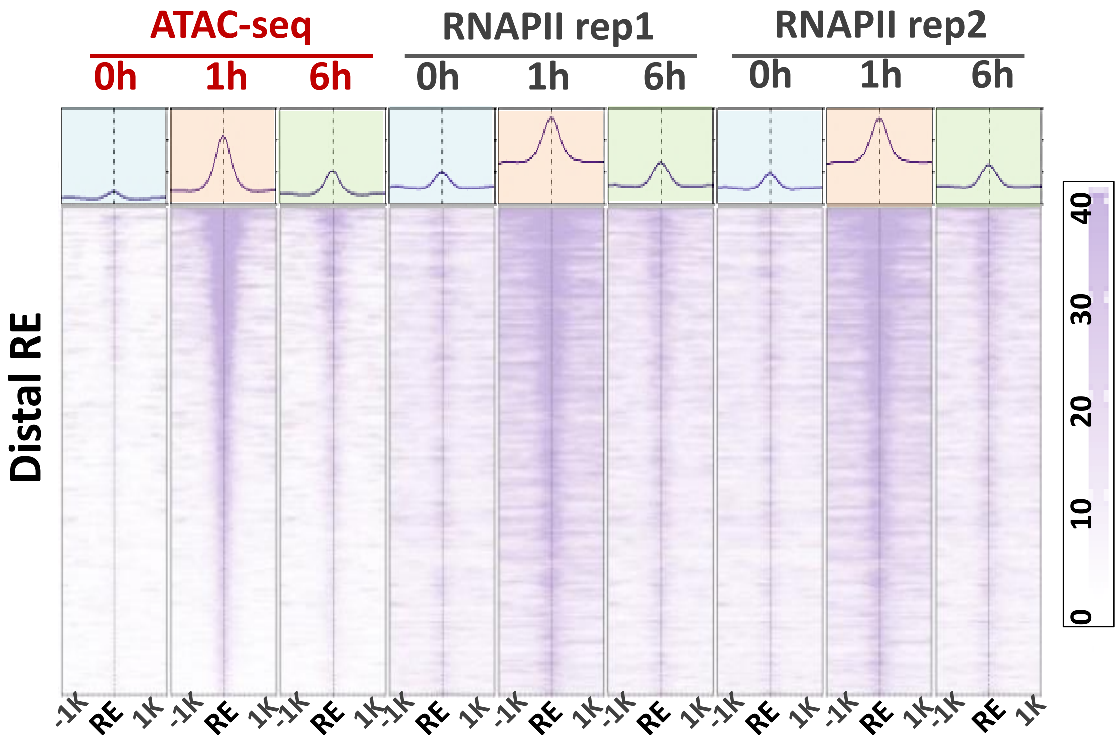

Supplementary Figure 14. Correlation between DNA accessibility and RNAPII binding in distal RE
Heatmap of ATAC-seq and ChIP-seq of the RNAPII profile at 0, 1, and 6 h of heat stress (2 replicates).

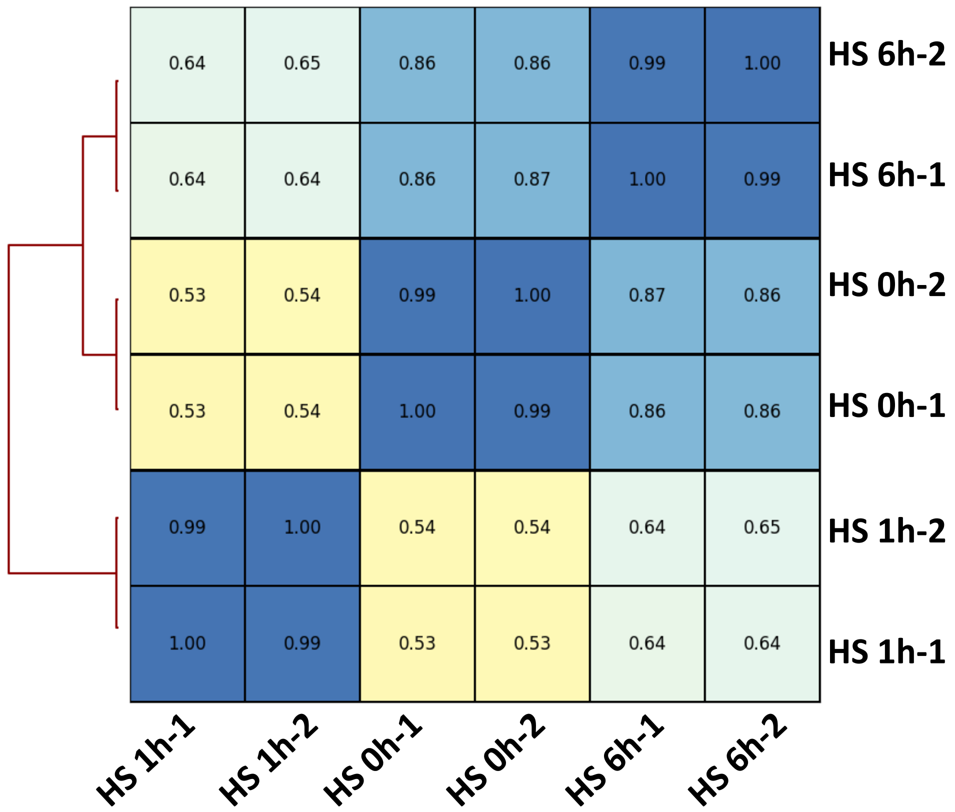

Supplementary Figure 15. Correlation between ChIP-seq RNAPII replicates after 0, 1, and 6 h of HS.

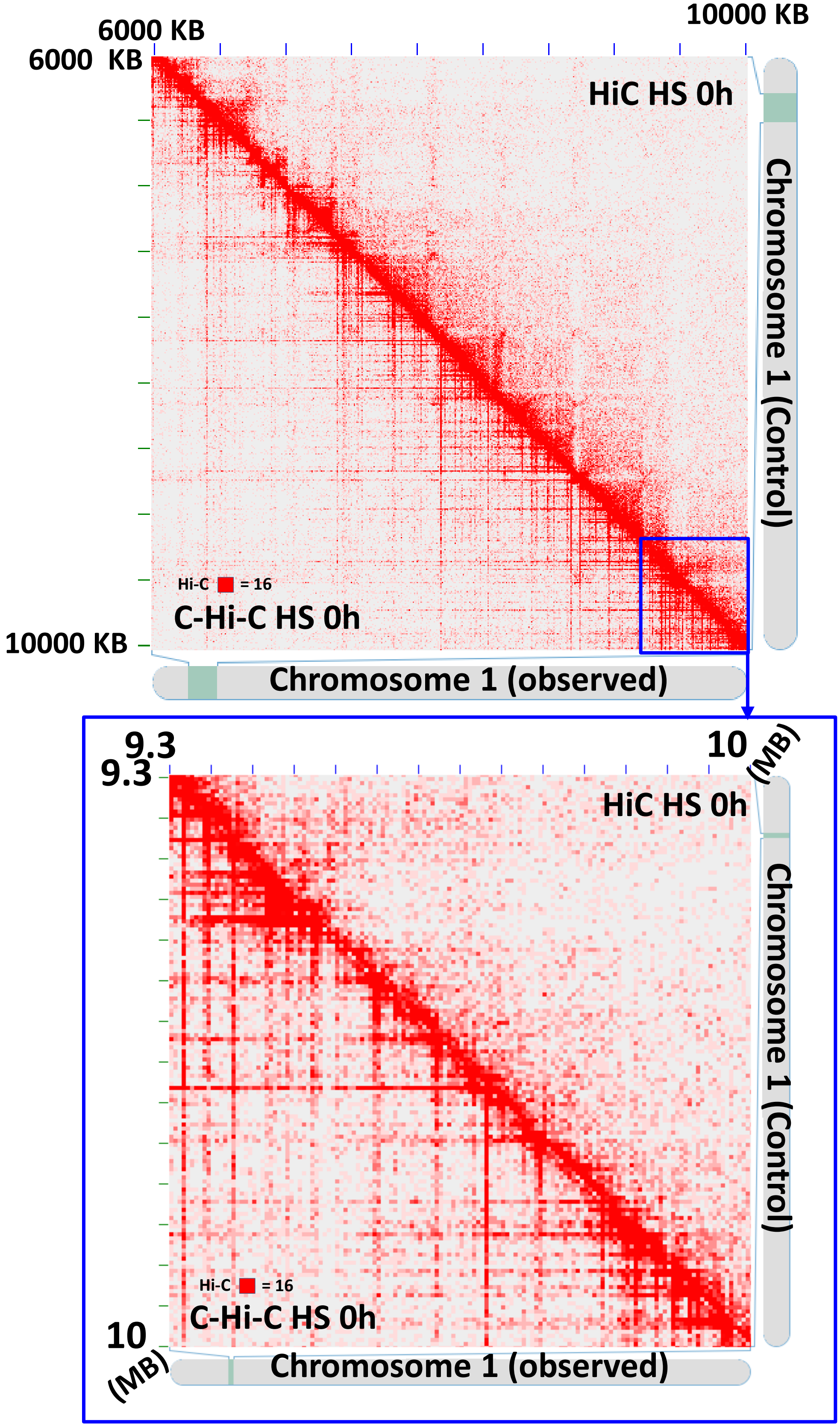

Supplementary Figure 16. Heatmap of C-Hi-C and Hi-C in the chromosome 1
Visualization of the interaction matrix of Hi-C and C-Hi-C in a specific region of the chromosome 1.

a

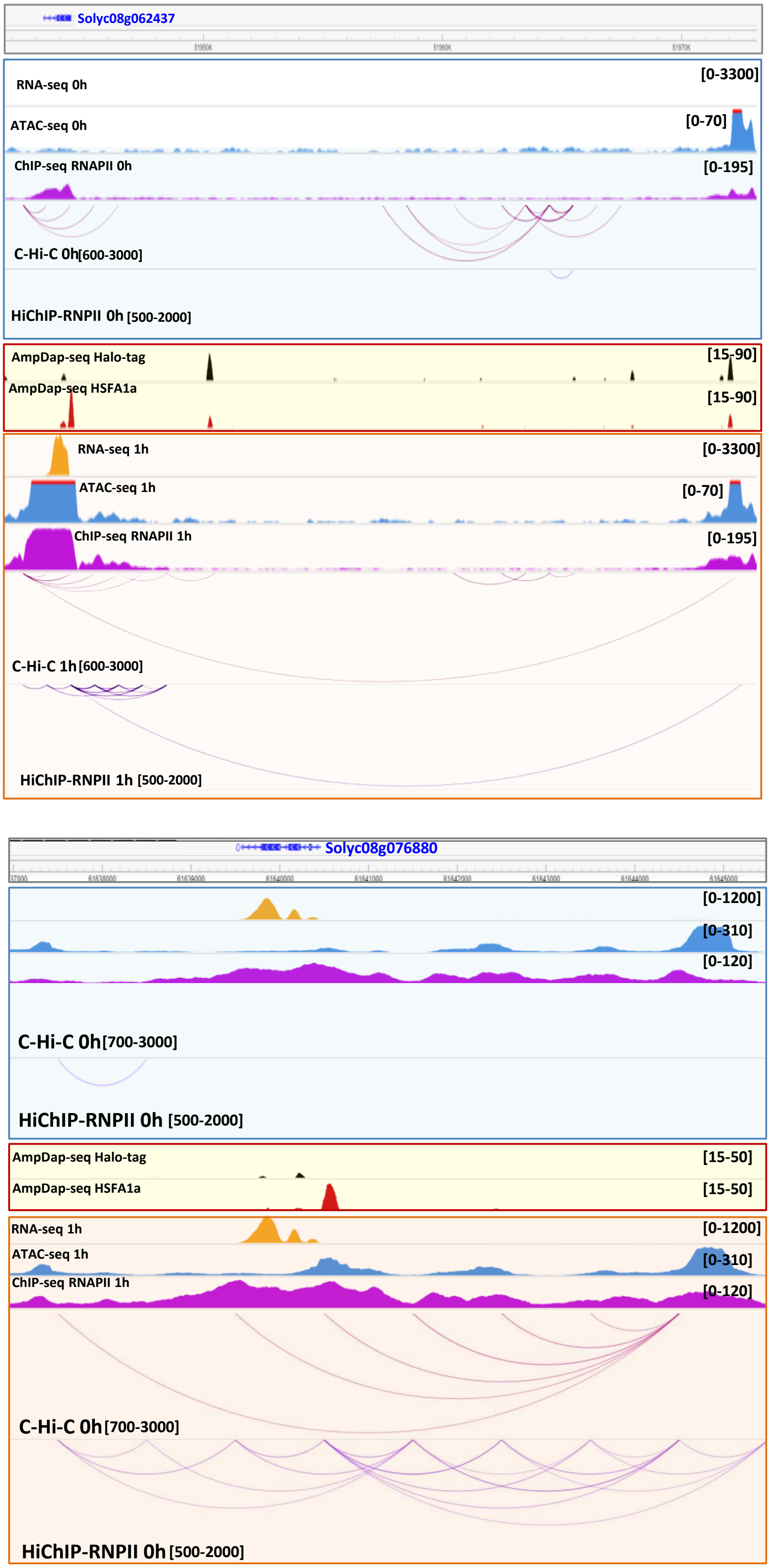

b

Supplementary Figure 17. Examples of promoter and enhancer hubs. Significant interactions for C-Hi-C and HiChIP-RNPII data were detected with HOMER (v4.11) using cumulative binomial distribution with p value 0.05.

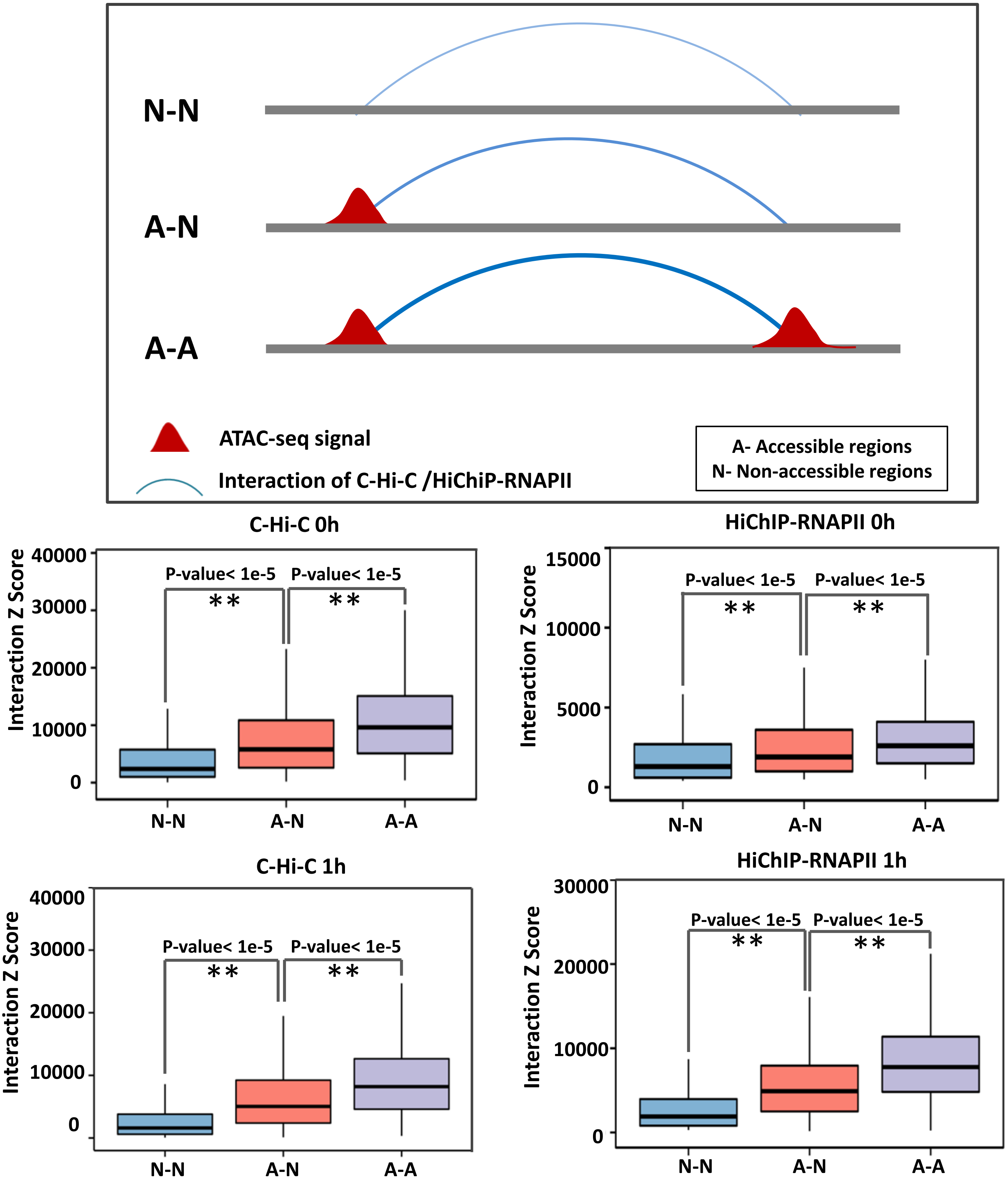

Supplementary Figure 18. RE- promoter chromatin interaction strength. Each feature was compared with t test (n1-C-HiC 0h=27007; n2-C-HiC 1h=24269; n1-C-HiC 0h=19779; n1-C-HiC 0h=23881) along with multiple comparisons and adjusted P values. For each box plot, center lines indicated the medians; boxes showed the 25th and 75th percentiles; outlier values have been removed. Source data of Supplementary Figure 13 are provided as a Source Data file. Source data are provided as a Source Data file.

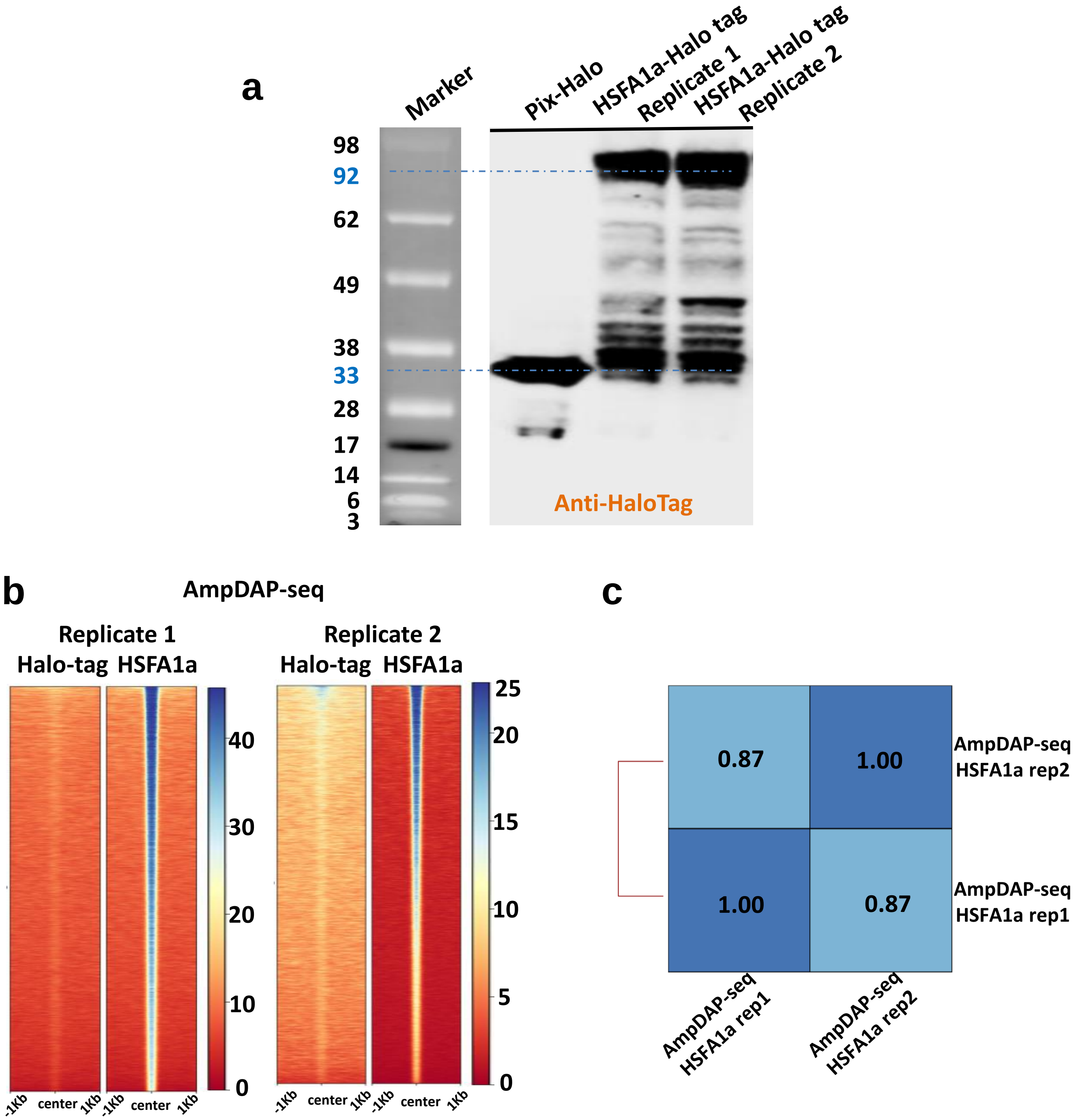

Supplementary Figure 19. The HSFA1a TF plays an important role in loop formation in response to heat stress

(a) Immunoblot of protein expression of Pix-halo and HSFA1a-Halo tag (two independent replicates).
(b) Heatmap of AmpDAP-seq of Halo-tag (control) and HSFA1a profiles (2 independent replicates).
(c) Correlation of the AmpDAP-seq HSFA1a between replicates after 0, 1, and 6 h of HS. 3 times each experiment was repeated independently with similar results. Source data of are provided as a Source Data file.

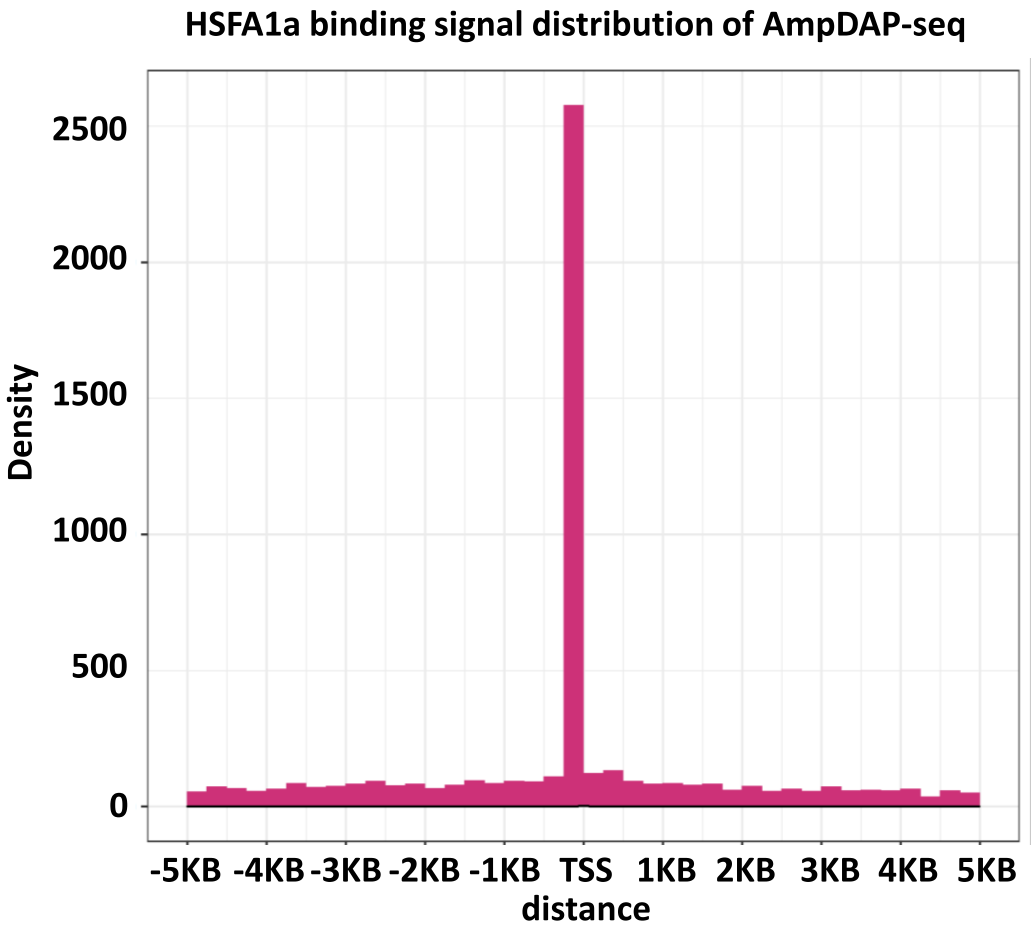

Supplementary Figure 20. HSFA1a binding signal distribution of AmpDAP-seq

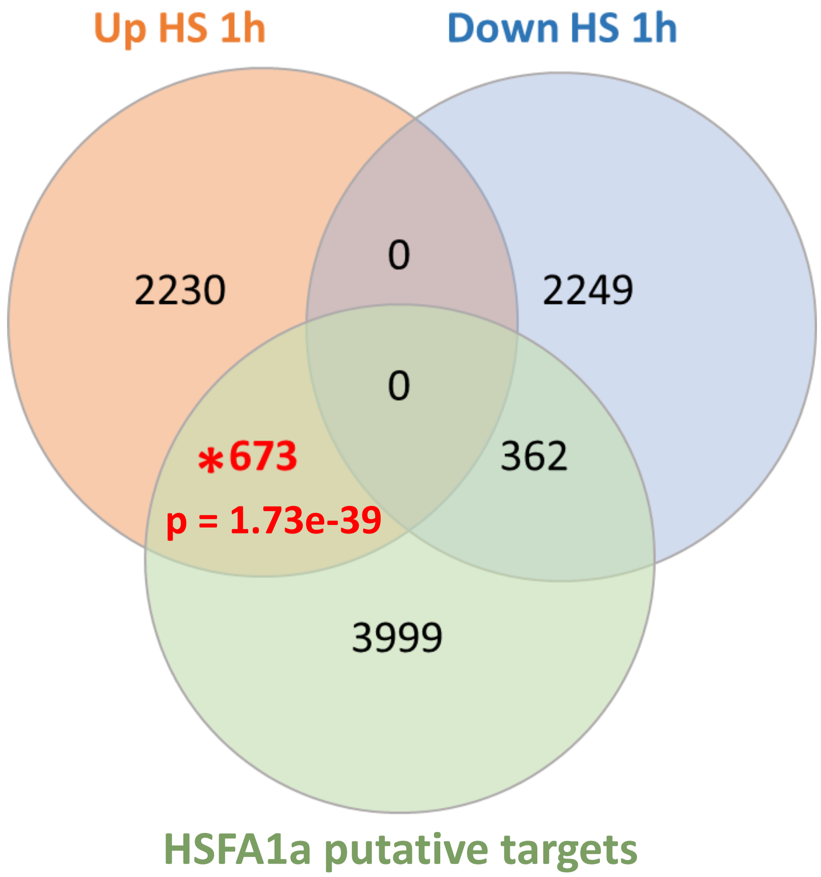

Supplementary Figure 21. Venn diagram showing the overlap between differentially expressed genes after 1h of heat stress and HSFA1a putative targets. P values (Fisher's exact test) for overlaps between gene sets are denoted. Source data are provided as a Source Data file.

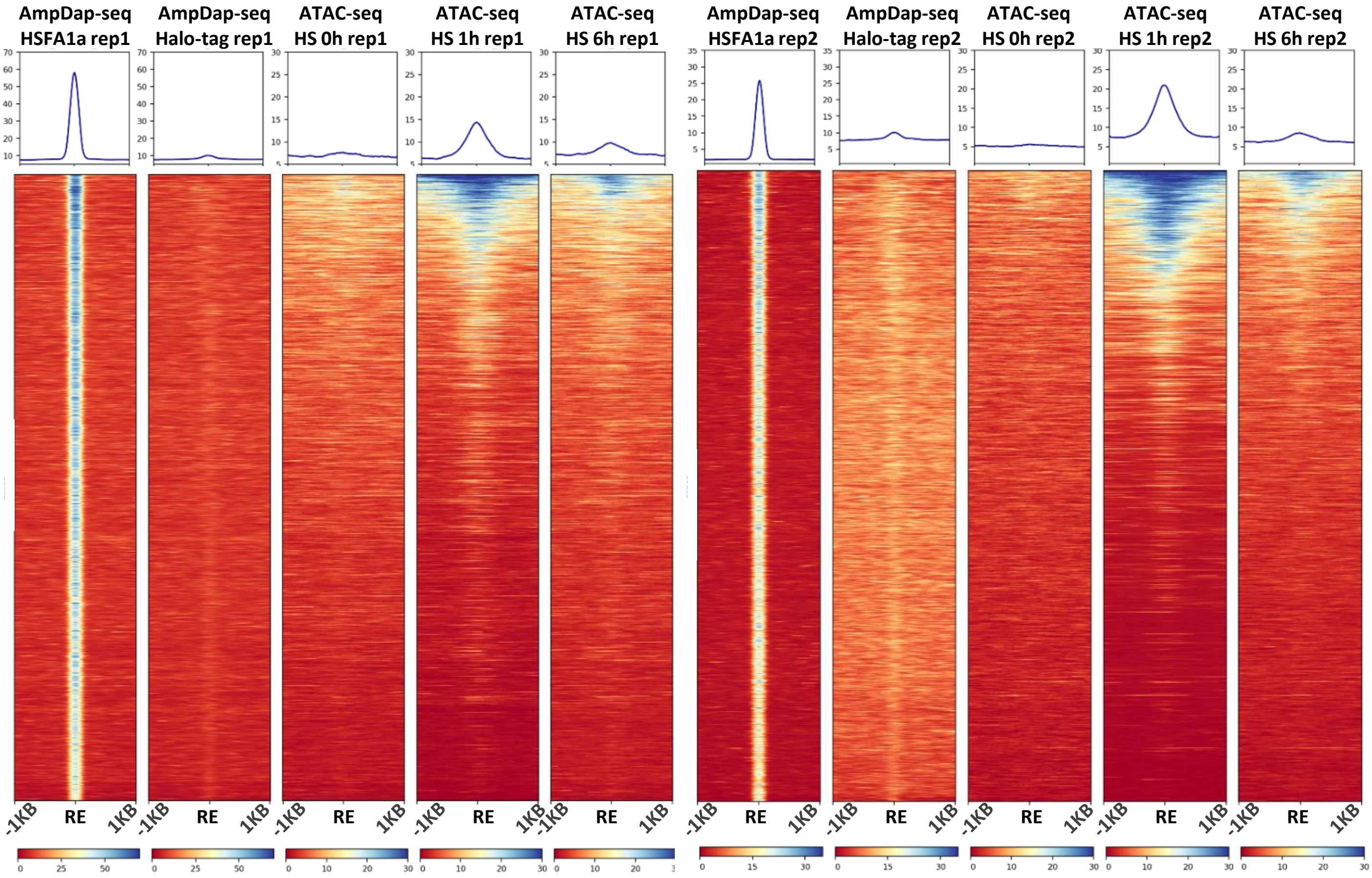

Supplementary Figure 22. Heatmaps of AmpDAP-seq HSFA1a and ATAC-seq profiles over HSFA1a targets.

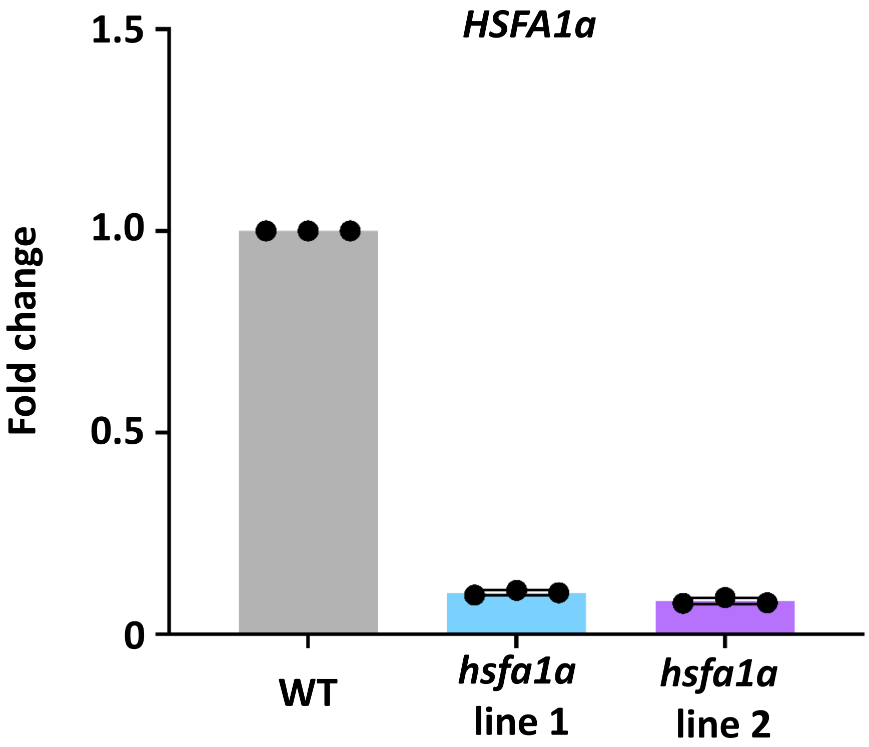

Supplementary Figure 23. ***HSFA1*** **expression level in** ***hsfa1a*** **knock-down lines.** β-actin was used to normalize. Data are presented as mean values +/- standard deviation (SD) from three biological replicates (n=3). (Two-sided Student's t-test, exact p-values are added on the bars). Source data are provided as a Source Data file.

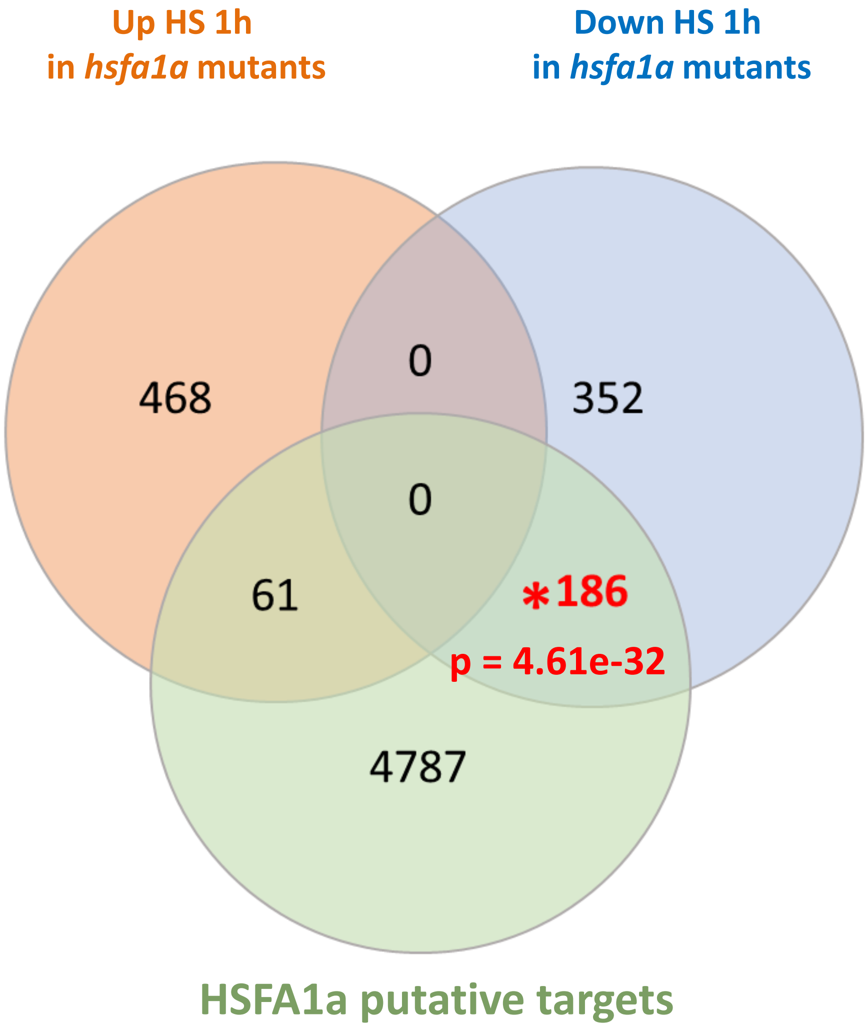

Supplementary Figure 24. Venn diagram showing the overlap between differentially expressed genes after 1 h of heat stress in hsfa1a knock down lines and HSFA1a putative targets. P values (Fisher's exact test) for overlaps between gene sets are denoted. Source data are provided as a Source Data file.

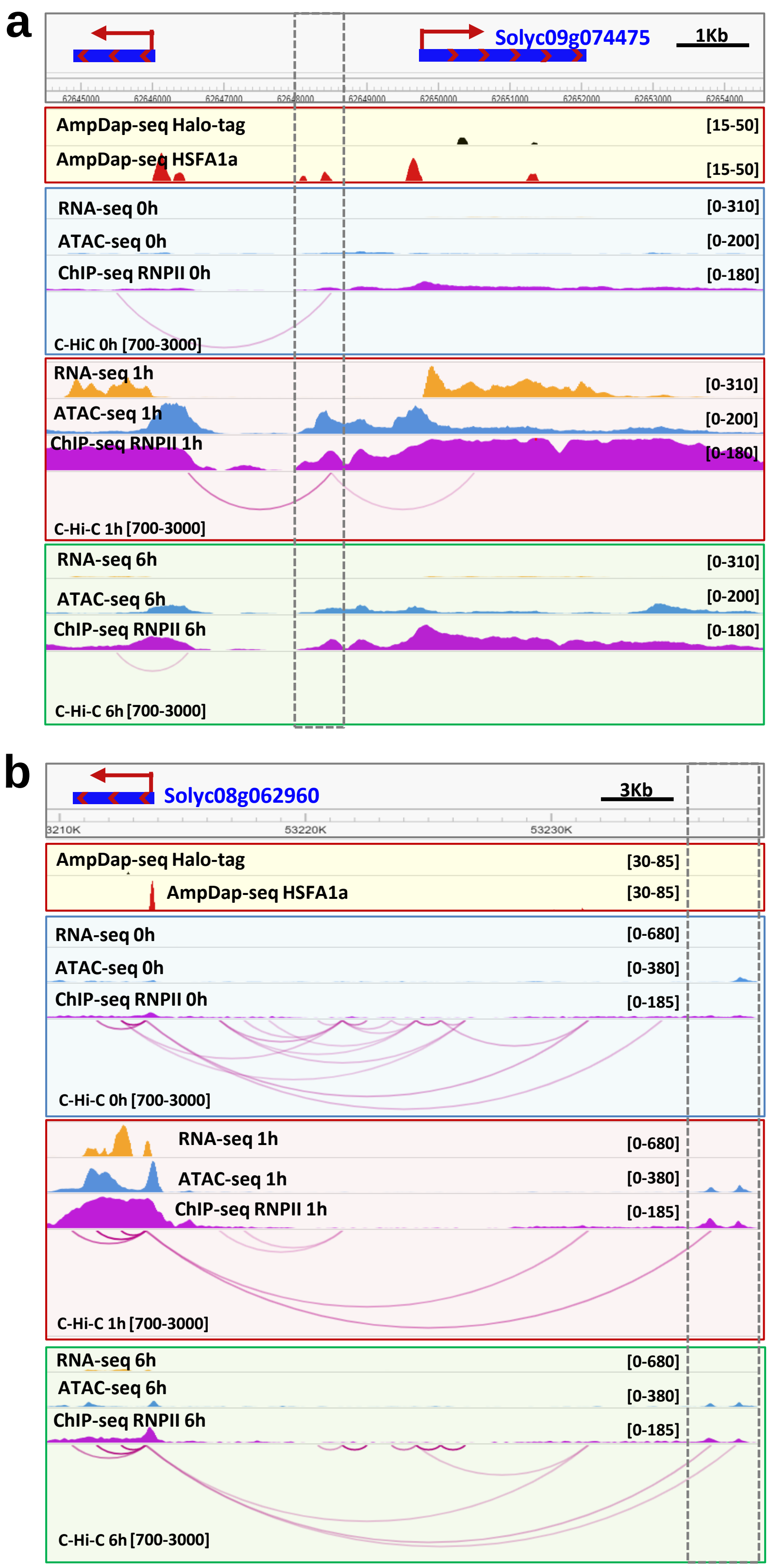

Supplementary Figure 25. Screenshot of the *Solyc09g074475* (a) and *HSFA2* (b) loci

DAP-seq against Halo-tag and HSFA1 are represented respectively in black and red. RNA-seq signal is represented in orange, ATAC-seq signal in blue, and RNAPII ChIP-seq in purple. Chromatin interactions from C-Hi-C data are represented by red lines. Significant interactions for C-Hi-C and HiChIP-RNPII data were detected with HOMER (v4.11) using cumulative binomial distribution with p value 0.05.

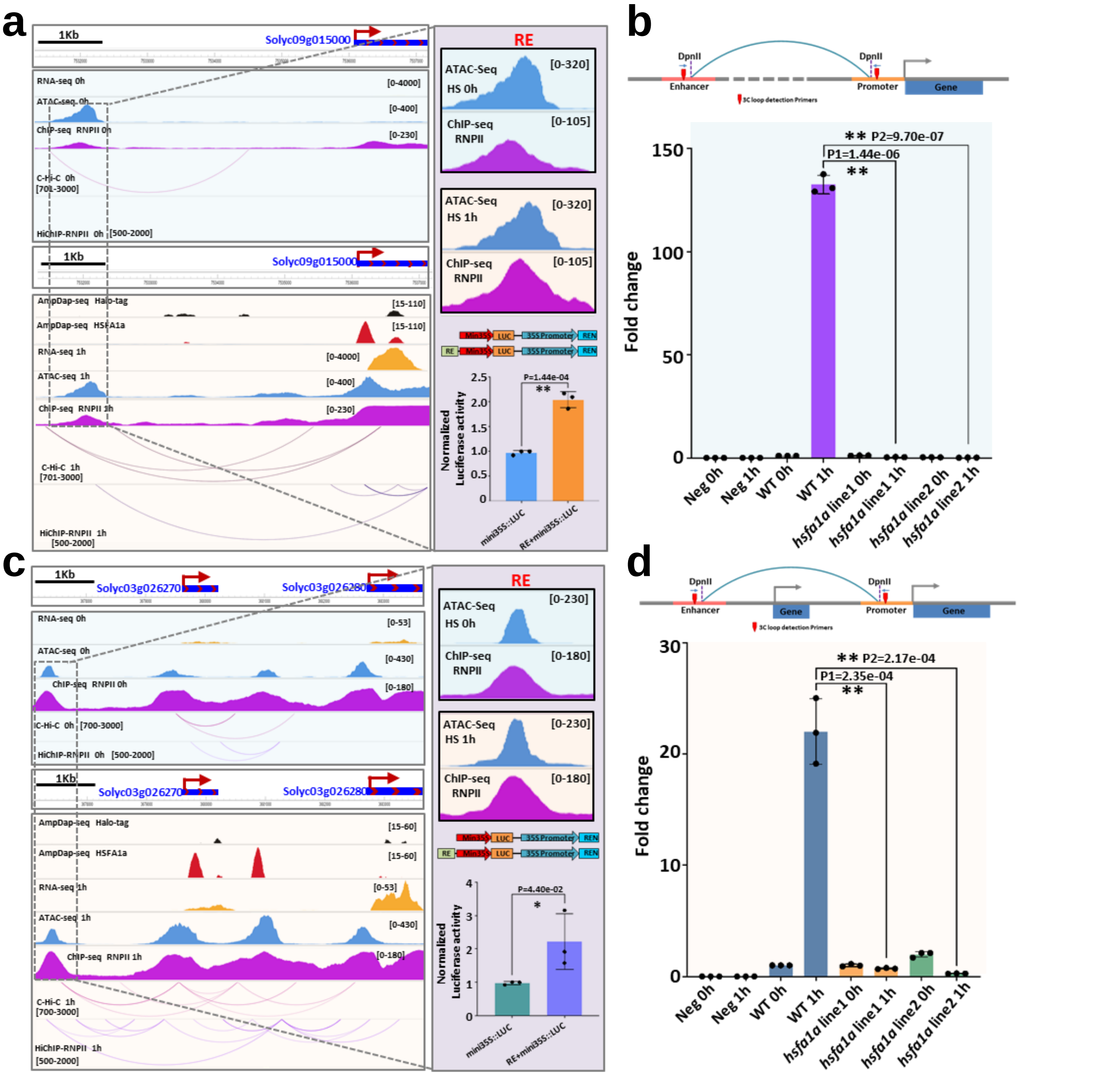

Supplementary Figure 26. Two examples of enhancer-promoter loops controlled by HSF1a in response to heat stress. (a)(c) DAP-seq against Halo-tag and HSFA1 are represented in black and red, respectively. The RNA-seq signal is represented in orange, the ATAC-seq signal in blue, and the RNAPII ChIP-seq signal in purple. Chromatin interactions from C-Hi-C data are represented by red lines and RNAPII HiChIP data by purple lines. Significant interactions for C-Hi-C and HiChIP-RNPII data were detected with HOMER (v4.11) using cumulative binomial distribution with p value 0.05. The right box shows a zoom of a distal RE that interacts with heat stress-responsive genes. The histogram represents the luciferase activity from a transient luciferase assay using a *RE-mini35S::LUC* construct or the *mini35S::LUC* alone as a control. The mean LUC/REN activity levels were normalized to mini35S::LUC as control (n = 3 biological replicates). The mean LUC/REN activity levels were normalized to mini35S::LUC as control (n = 3 biological replicates). Circles denote relative LUC/REN activity values. Bars indicate the mean values +/- SD of three biological replicates. Exact P values are shown (two-tailed, two-sample Student's t-test).**(b)(d)** (Top) Design of the 3C-qPCR assays used to analyze the distal RE loops in the WT and hsfa1a lines in response to heat stress. (Bottom) Quantitative 3C; the relative interaction frequencies were calculated as described in the Materials and Methods. Data are the average of three biological replicates each with three technical replicates (n=3). Circles denote relative interaction frequency. Bars indicate mean values +/- SD of three replicates. Exact P values are shown (two-tailed, two-sample Student's t-test). Source data are provided as a Source Data file.
