# Supplementary material for: HSFA1a modulates plant heat stress responses and alters the 3D chromatin organization of enhancer-promoter interactions
Source: Nat Commun. 2023 Jan 28;14:469. doi: 10.1038/s41467-023-36227-3 (PMC9884265; doi:10.1038/s41467-023-36227-3)
Supplement: Supplementary file 2 — Description of additional Supplementary File [file 41467_2023_36227_MOESM2_ESM.pdf]

Descriptions of additional supplementary files

Supplementary Data 1: Probes details for Capture-Hi-C experiments.

Supplementary Data 2: Data from bioinformatic analysis.

Supplementary Data 3: Primers used in this study

Supplementary Data 4: Sequencing data information
