# Supplementary material for: HSFA1a modulates plant heat stress responses and alters the 3D chromatin organization of enhancer-promoter interactions
Source: Nat Commun. 2023 Jan 28;14:469. doi: 10.1038/s41467-023-36227-3 (PMC9884265; doi:10.1038/s41467-023-36227-3)
Supplement: Supplementary file 7 — Reporting Summary [file 41467_2023_36227_MOESM7_ESM.pdf]

nature portfolio

Corresponding author(s): Mossa Benhamed

Last updated by author(s): Dec 31, 2022

Reporting Summary

Nature Portfolio wishes to improve the reproducibility of the work that we publish. This form provides structure for consistency and transparency in reporting. For further information on Nature Portfolio policies, see our Editorial Policies and the Editorial Policy Checklist.

Statistics

For all statistical analyses, confirm that the following items are present in the figure legend, table legend, main text, or Methods section.

n/a	Confirmed	
☐	☒	The exact sample size (*n*) for each experimental group/condition, given as a discrete number and unit of measurement
☐	☒	A statement on whether measurements were taken from distinct samples or whether the same sample was measured repeatedly
☐	☒	The statistical test(s) used AND whether they are one- or two-sided<br>*Only common tests should be described solely by name; describe more complex techniques in the Methods section.*
☒	☐	A description of all covariates tested
☐	☒	A description of any assumptions or corrections, such as tests of normality and adjustment for multiple comparisons
☐	☒	A full description of the statistical parameters including central tendency (e.g. means) or other basic estimates (e.g. regression coefficient) AND variation (e.g. standard deviation) or associated estimates of uncertainty (e.g. confidence intervals)
☐	☒	For null hypothesis testing, the test statistic (e.g. *F*, *t*, *r*) with confidence intervals, effect sizes, degrees of freedom and *P* value noted<br>*Give P values as exact values whenever suitable.*
☒	☐	For Bayesian analysis, information on the choice of priors and Markov chain Monte Carlo settings
☐	☒	For hierarchical and complex designs, identification of the appropriate level for tests and full reporting of outcomes
☐	☒	Estimates of effect sizes (e.g. Cohen's *d*, Pearson's *r*), indicating how they were calculated

Our web collection on statistics for biologists contains articles on many of the points above.

Software and code

Policy information about availability of computer code

Data collection	No software was used during data collection.
Data analysis	trimmomatic-0.38.jar, bowtie2 v 2.3.5, macs2 v2.2.7.1, s3norm v 2, HiC-Pro v 2.11.4, featureCounts v 2.0.0, R 3.5.1(www.R-project.org), DESeq2 v 1.38.0, HOMER (v4.11).

For manuscripts utilizing custom algorithms or software that are central to the research but not yet described in published literature, software must be made available to editors and reviewers. We strongly encourage code deposition in a community repository (e.g. GitHub). See the Nature Portfolio guidelines for submitting code & software for further information.

Data

Policy information about availability of data

All manuscripts must include a data availability statement. This statement should provide the following information, where applicable:

- Accession codes, unique identifiers, or web links for publicly available datasets
- A description of any restrictions on data availability
- For clinical datasets or third party data, please ensure that the statement adheres to our policy

Raw data have been deposited to the Gene Expression Omnibus (GEO) database under accession number GSE206365.

Human research participants

Policy information about studies involving human research participants and Sex and Gender in Research.

Reporting on sex and gender	N/A
Population characteristics	N/A
Recruitment	N/A
Ethics oversight	N/A

Note that full information on the approval of the study protocol must also be provided in the manuscript.

Field-specific reporting

Please select the one below that is the best fit for your research. If you are not sure, read the appropriate sections before making your selection.

[x] Life sciences [ ] Behavioural & social sciences [ ] Ecological, evolutionary & environmental sciences

For a reference copy of the document with all sections, see nature.com/documents/nr-reporting-summary-flat.pdf

Life sciences study design

All studies must disclose on these points even when the disclosure is negative.

Sample size	No sample-size calculations were performed. Sample size was determined to be adequate based on the consistency of measurements. Seeds were directly sown on soil and plants grown in growth chambers at 24 °C under long-day (16 h light) conditions. For heat treatment, 4-week-old plants were treated at 45°C for 1 h and 6 h in a climatic chamber (Aralab). The fourth leaf was used for all experiments in this research.
Data exclusions	No data exclusions.
Replication	For ATAC-seq, RNA-seq, In situ Hi-C , C-Hi-C, ChIP-seq, AmpDAP-seq and 3C-qPCR assays, two independent biological replicates were generated.
Randomization	Biological replicates were randomly set. Each biological replicates consisted of randomly pooled seedlings. All the plants were grown under the same condition as described in the Methods. Seeds were directly sown on soil and plants grown in growth chambers at 24 °C under long-day (16 h light) conditions. For heat treatment, 4-week-old plants were treated at 45°C for 1 h and 6 h in a climatic chamber (Aralab). The fourth leaf was used for all experiments in this research.
Blinding	Investigators were not blinded to tomato under different conditions during experiments. Data reported are not subjective.

Reporting for specific materials, systems and methods

We require information from authors about some types of materials, experimental systems and methods used in many studies. Here, indicate whether each material, system or method listed is relevant to your study. If you are not sure if a list item applies to your research, read the appropriate section before selecting a response.

Materials & experimental systems

n/a	Involved in the study	
[ ]	[x]	Antibodies
[x]	[ ]	Eukaryotic cell lines
[x]	[ ]	Palaeontology and archaeology
[x]	[ ]	Animals and other organisms
[x]	[ ]	Clinical data
[x]	[ ]	Dual use research of concern

Methods

n/a	Involved in the study	
[ ]	[x]	ChIP-seq
[x]	[ ]	Flow cytometry
[x]	[ ]	MRI-based neuroimaging

Antibodies

Antibodies used	H3K9me2 (Abcam, ab1220), H3K27me1 (Millipore, 07-448), H3K9ac (Millipore, 07-352), H3K18ac (Millipore, 07-354), H3K27ac (Abcam, ab4729), H3K4me3 (Millipore, 07-473), RNAPII (Abcam, ab26721)

Validation

The antibodies used in this study have been validated by the manufacturer and we have extensively published papers (Huang et. al. 2021, Antunez-Sanchez et. al. 2020, Kim et. al. 2020, Concia et. al. 2020.) . In this study, these marks were validate and present in Fig.1, Fig.3 and Sup.Fig 9.

ChIP-seq

Data deposition

☒ Confirm that both raw and final processed data have been deposited in a public database such as GEO.

☒ Confirm that you have deposited or provided access to graph files (e.g. BED files) for the called peaks.

Data access links
May remain private before publication.

Raw data have been deposited to the Gene Expression Omnibus (GEO) database under accession number GSE206365.

Files in database submission

M82_0h_H3K4me3_rep1_S1_R1_001.fastq.gz
M82_0h_H3K4me3_rep2_S9_R1_001.fastq.gz
M82_1h_H3K4me3_rep1_S2_R1_001.fastq.gz
M82_1h_H3K4me3_rep2_S10_R1_001.fastq.gz
M82_6h_H3K4me3_rep1_S3_R1_001.fastq.gz
M82_6h_H3K4me3_rep2_S11_R1_001.fastq.gz
M82_0h_H3K9ac_rep1_S4_R1_001.fastq.gz
M82_1h_H3K9ac_rep1_S5_R1_001.fastq.gz
M82_6h_H3K9ac_rep1_S6_R1_001.fastq.gz
M82_0h_H3K9ac_rep2_S4_R1_001.fastq.gz
M82_1h_H3K9ac_rep2_S5_R1_001.fastq.gz
M82_6h_H3K9ac_rep2_S6_R1_001.fastq.gz
M82_0h_H3K18ac_rep1_S2_R1_001.fastq.gz
M82_1h_H3K18ac_rep1_S7_R1_001.fastq.gz
M82_6h_H3K18ac_rep1_S8_R1_001.fastq.gz
M82_0h_H3K18ac_rep2_S2_R1_001.fastq.gz
M82_1h_H3K18ac_rep2_S7_R1_001.fastq.gz
M82_6h_H3K18ac_rep2_S8_R1_001.fastq.gz
M82_0h_H3K27ac_rep1_S7_R1_001.fastq.gz
M82_1h_H3K27ac_rep1_S8_R1_001.fastq.gz
M82_6h_H3K27ac_rep1_S9_R1_001.fastq.gz
M82_0h_H3K27ac_rep2_S7_R1_001.fastq.gz
M82_1h_H3K27ac_rep2_S8_R1_001.fastq.gz
M82_6h_H3K27ac_rep2_S9_R1_001.fastq.gz
M82_0h_Pol2_rep1_S10_R1_001.fastq.gz
M82_0h_Pol2_rep2_S5_R1_001.fastq.gz
M82_1h_Pol2_rep1_S6_R1_001.fastq.gz
M82_1h_Pol2_rep2_S7_R1_001.fastq.gz
M82_6h_Pol2_rep1_S8_R1_001.fastq.gz
M82_6h_Pol2_rep2_S9_R1_001.fastq.gz
M82_0h_H3K27me1_0h_rep1.fastq.gz
M82_Input_S12_R1_001.fastq.gz
M82_0h_H3K4me3_rep1_p0.05_peaks.narrowPeak.gz
M82_0h_H3K4me3_rep1_s3norm.bigwig
M82_0h_H3K4me3_rep2_p0.05_peaks.narrowPeak.gz
M82_0h_H3K4me3_rep2_s3norm.bigwig
M82_1h_H3K4me3_rep1_p0.05_peaks.narrowPeak.gz
M82_1h_H3K4me3_rep1_s3norm.bigwig
M82_1h_H3K4me3_rep2_p0.05_peaks.narrowPeak.gz
M82_1h_H3K4me3_rep2_s3norm.bigwig
M82_6h_H3K4me3_rep1_p0.05_peaks.narrowPeak.gz
M82_6h_H3K4me3_rep1_s3norm.bigwig
M82_6h_H3K4me3_rep2_p0.05_peaks.narrowPeak.gz
M82_6h_H3K4me3_rep2_s3norm.bigwig
M82_0h_H3K9ac_rep1_p0.05_peaks.narrowPeak.gz
M82_0h_H3K9ac_rep1_s3norm.bigwig
M82_1h_H3K9ac_rep1_p0.05_peaks.narrowPeak.gz
M82_1h_H3K9ac_rep1_s3norm.bigwig
M82_6h_H3K9ac_rep1_p0.05_peaks.narrowPeak.gz
M82_6h_H3K9ac_rep1_s3norm.bigwig
M82_0h_H3K9ac_rep2_p0.05_peaks.narrowPeak.gz
M82_0h_H3K9ac_rep2_s3norm.bigwig
M82_1h_H3K9ac_rep2_p0.05_peaks.narrowPeak.gz
M82_1h_H3K9ac_rep2_s3norm.bigwig

M82_6h_H3K9ac_rep2_p0.05_peaks.narrowPeak.gz
M82_6h_H3K9ac_rep2_s3norm.bigwig
M82_0h_H3K18ac_rep1_p0.05_peaks.narrowPeak.gz
M82_0h_H3K18ac_rep1_s3norm.bigwig
M82_1h_H3K18ac_rep1_p0.05_peaks.narrowPeak.gz
M82_1h_H3K18ac_rep1_s3norm.bigwig
M82_6h_H3K18ac_rep1_p0.05_peaks.narrowPeak.gz
M82_6h_H3K18ac_rep1_s3norm.bigwig
M82_0h_H3K18ac_rep2_p0.05_peaks.narrowPeak.gz
M82_0h_H3K18ac_rep2_s3norm.bigwig
M82_1h_H3K18ac_rep2_p0.05_peaks.narrowPeak.gz
M82_1h_H3K18ac_rep2_s3norm.bigwig
M82_6h_H3K18ac_rep2_p0.05_peaks.narrowPeak.gz
M82_6h_H3K18ac_rep2_s3norm.bigwig
M82_0h_H3K27ac_rep1_p0.05_peaks.narrowPeak.gz
M82_0h_H3K27ac_rep1_s3norm.bigwig
M82_1h_H3K27ac_rep1_p0.05_peaks.narrowPeak.gz
M82_1h_H3K27ac_rep1_s3norm.bigwig
M82_6h_H3K27ac_rep1_p0.05_peaks.narrowPeak.gz
M82_6h_H3K27ac_rep1_s3norm.bigwig
M82_0h_H3K27ac_rep2_p0.05_peaks.narrowPeak.gz
M82_0h_H3K27ac_rep2_s3norm.bigwig
M82_1h_H3K27ac_rep2_p0.05_peaks.narrowPeak.gz
M82_1h_H3K27ac_rep2_s3norm.bigwig
M82_6h_H3K27ac_rep2_p0.05_peaks.narrowPeak.gz
M82_6h_H3K27ac_rep2_s3norm.bigwig
M82_0h_Pol2_rep1_p0.05_peaks.narrowPeak.gz
M82_0h_Pol2_rep1_s3norm.bigwig
M82_0h_Pol2_rep2_p0.05_peaks.narrowPeak.gz
M82_0h_Pol2_rep2_s3norm.bigwig
M82_1h_Pol2_rep1_p0.05_peaks.narrowPeak.gz
M82_1h_Pol2_rep1_s3norm.bigwig
M82_1h_Pol2_rep2_p0.05_peaks.narrowPeak.gz
M82_1h_Pol2_rep2_s3norm.bigwig
M82_6h_Pol2_rep1_p0.05_peaks.narrowPeak.gz
M82_6h_Pol2_rep1_s3norm.bigwig
M82_6h_Pol2_rep2_p0.05_peaks.narrowPeak.gz
M82_6h_Pol2_rep2_s3norm.bigwig

Genome browser session (e.g. UCSC)

N/A

Methodology

Replicates

Two independent biological replicates were generated of ChIP-seq.

Sequencing depth

All ChIP-seq DNA libraries were subjected to 1×75bp high-throughput sequencing by NextSeq 500 (Illumina).

Antibodies

H3K27me1 (Millipore, 07-448), H3K9ac (Millipore, 07-352), H3K18ac (Millipore, 07-354), H3K27ac (Abcam, ab4729), H3K4me3 (Millipore, 07-473), RNAPII (Abcam, ab26721)

Peak calling parameters

Peak calling: peaks of read density were called with macs2 2.2.7.1 with the command "macs2 callpeak -t sample.bam -c Input.bam -g 829069930 -p 0.05 --extsize 150 --bw 500 -B -n --outdir

Data quality

Adapters trimming: Sequencing reads were trimmed with trimmomatic with the following command "java -jar trimmomatic-0.38.jar SE $input $output ILLUMINACLIP:TruSeq3-SE.fa:2:30:10 LEADING:5 TRAILING:5 MINLEN:30"
Filtering step: Mapped reads were filtered with samtools v.1.9 with the command "samtools view -h -b -q 30 "mapping_quality >= 30"
Duplicate filtering: duplicated reads were removed with samtools v.1.9 with the command "samtools fixmate -m and samtools markdup -r "

Software

trimmomatic-0.38.jar, bowtie2 v 2.3.5, macs2 2.2.7.1, s3norm
